# FOXO1 pathway activation by VISTA immune checkpoint restrains pulmonary ILC2 functions

**DOI:** 10.1172/JCI184932

**Published:** 2025-01-02

**Authors:** Mohammad Hossein Kazemi, Zahra Momeni-Varposhti, Xin Li, Benjamin P. Hurrell, Yoshihiro Sakano, Stephen Shen, Pedram Shafiei-Jahani, Kei Sakano, Omid Akbari

**Affiliations:** Department of Molecular Microbiology and Immunology, Keck School of Medicine, University of Southern California, Los Angeles, California, USA.

**Keywords:** Immunology, Inflammation, Allergy, Asthma, Innate immunity

## Abstract

Group 2 innate lymphoid cells (ILC2s) play a pivotal role in the development of airway hyperreactivity (AHR). However, the regulatory mechanisms governing ILC2 function remain inadequately explored. This study uncovers V-domain Ig suppressor of T cell activation (VISTA) as an inhibitory immune checkpoint crucial for modulating ILC2-driven lung inflammation. VISTA is upregulated in activated pulmonary ILC2s and plays a key role in regulating lung inflammation, as VISTA-deficient ILC2s demonstrate increased proliferation and function, resulting in elevated type 2 cytokine production and exacerbation of AHR. Mechanistically, VISTA stimulation activates Forkhead box O1 (FOXO1), leading to modulation of ILC2 proliferation and function. The suppressive effects of FOXO1 on ILC2 effector function were confirmed using FOXO1 inhibitors and activators. Moreover, VISTA-deficient ILC2s exhibit enhanced fatty acid oxidation and oxidative phosphorylation to meet their high energy demands. Therapeutically, VISTA agonist treatment reduces ILC2 function both ex vivo and in vivo, significantly alleviating ILC2-driven AHR. Our murine findings were validated in human ILC2s, whose function was reduced ex vivo by a VISTA agonist, and in a humanized mouse model of ILC2-driven AHR. Our studies unravel VISTA as an immune checkpoint for ILC2 regulation via the FOXO1 pathway, presenting potential therapeutic strategies for allergic asthma by modulating ILC2 responses.

## Abstract 2

VISTA regulates ILC2 function via the FOXO1 pathway. VISTA agonists reduce ILC2-driven airway hyperreactivity, indicating potential therapy for allergic asthma.

## Introduction

Allergic asthma is a respiratory condition characterized by airway hyperreactivity (AHR), inflammation, and remodeling (1). Recent discoveries have shed light on the pivotal contributions of innate immune cells, particularly group 2 innate lymphoid cells (ILC2s). ILC2s are key orchestrators of type 2 immune responses, involved in initiating and perpetuating AHR independently of the adaptive immune system (2, 3). ILC2s are the predominant ILC population in the lungs at steady state and possess a unique ability to rapidly respond to danger signals, such as interleukin-25 (IL-25), IL-33, and thymic stromal lymphopoietin (TSLP), by producing type 2 cytokines including IL-5 and IL-13 (3). This immediate response leads to the recruitment of eosinophils to the airways and the development of AHR, hallmark features of allergic asthma (3, 4).

Despite similarities between ILC2s and Th2 cells in their effector function, the regulatory mechanisms governing ILC2 function, particularly in the context of allergic AHR, are yet to be fully investigated. Recent advances unraveled the complex regulatory networks governing ILC2 function (4). Understanding these regulatory networks is crucial for developing targeted therapies to modulate their function and improve asthma management. In this context, immune checkpoints play critical roles in fine-tuning immune responses and maintaining immune homeostasis. Several immune checkpoints have been identified to regulate ILC2 responses through various mechanisms (3, 5–8).

V-domain Ig suppressor of T cell activation (VISTA), also known as PD-1H, is a novel inhibitory immune checkpoint that has garnered significant attention for its multifaceted roles in immune regulation (9). VISTA is a member of the B7 family exhibiting structural and functional similarities to PD-1 but has distinct signaling pathways and a non-redundant effect on immune cells (10). VISTA intracellular domain contains a conserved Src homology 2 (SH2) binding motif and SH3 binding domains, which may serve as potential sites for initiating downstream signaling cascades (9, 11). VISTA was initially identified on naive T lymphocytes as a critical checkpoint regulator in maintaining peripheral T cell tolerance (9). While VISTA ligands are not yet fully characterized, recent studies showed that V-set and immunoglobulin domain containing 3 (VSIG3) and CD162 can bind to VISTA (12, 13). Among these two, only VSIG3 binds to VISTA at the physiological pH (13, 14), as a recent report shows that VSIG3 binding to T cells via VISTA inhibits proliferation and effector function (12). Further evidence has implicated VISTA in modulating immune responses beyond T cell regulation. VISTA expression on various myeloid and lymphoid cells reprograms these cells toward an antiinflammatory phenotype, thereby dampening excessive immune responses and promoting immune tolerance (11, 15, 16). Genetic deletion or blocking of VISTA has been associated with various autoimmune and inflammatory conditions (9, 17–19). Conversely, agonist anti-VISTA monoclonal antibodies have shown promise in attenuating immune responses and alleviating the severity of inflammatory diseases (17, 20). However, the role of VISTA in innate lymphoid cells had not been investigated yet.

In this study, we uncover the regulatory role of VISTA in lung ILC2 function and its potential as a therapeutic target to modulate ILC2-mediated AHR and lung inflammation. We observed that naive pulmonary ILC2s express VISTA, and this expression is increased following activation. Notably, ILC2s lacking VISTA demonstrate increased proliferation, activation, and type 2 cytokine production, accompanied by an increase in metabolic pathways such as tricarboxylic acid (TCA) cycle, fatty acid oxidation (FAO), and oxidative phosphorylation (OXPHOS), resulting in greater oxygen consumption. This hyperactivation of ILC2s exacerbates ILC2-driven AHR and lung inflammation in VISTA-deficient mice. We validated the impact of VISTA deficiency solely on ILC2s through experiments using an adoptive transfer model. Conversely, treatment with a VISTA agonist suppresses ILC2 proliferation and effector function, alleviating AHR and lung inflammation in models of ILC2-dependent AHR. Mechanistically, VISTA activation upregulates Forkhead box O1 (FOXO1) transcription factor and reduces both canonical and non-canonical NF-κB signaling, which reduces ILC2 responses. Treatment of ILC2s with a FOXO1 inhibitor enhances ILC2 activity. Conversely, a FOXO1 activator potently suppresses ILC2 effector function. In a translational approach, we confirmed VISTA expression on human ILC2s and showed that agonizing VISTA in human ILC2s reduces ILC2 function ex vivo and also in a humanized mouse model of ILC2-driven AHR.

Collectively, our findings introduce VISTA as an immune checkpoint for ILC2 regulation, acting through the FOXO1 pathway. Understanding the intricate interplay between immune checkpoints and ILC2 responses holds promise for unraveling the pathogenesis of allergic asthma and developing targeted immunotherapies.

## Results

### VISTA is induced in pulmonary ILC2s upon activation.

**Figure 1:**
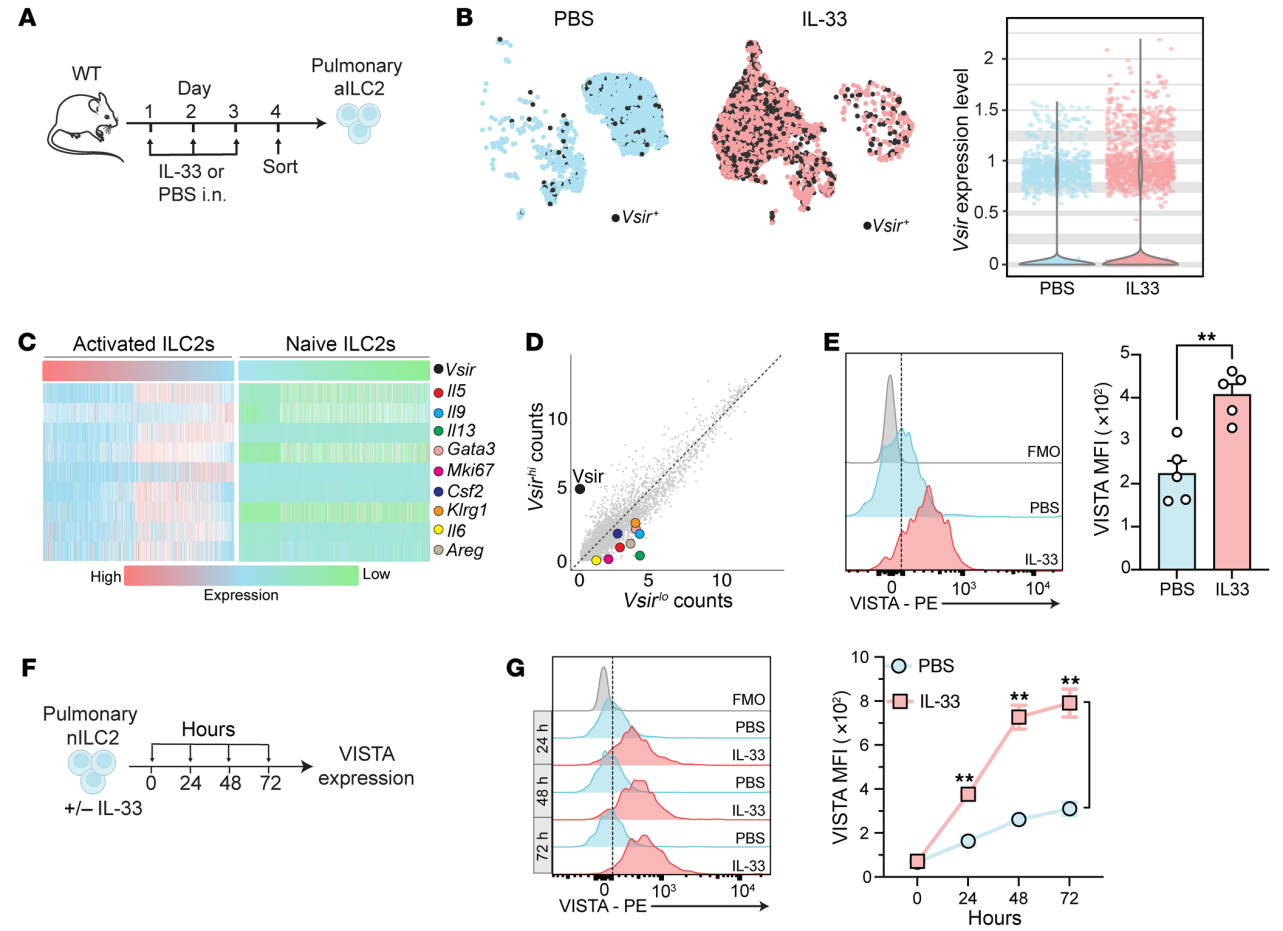
ILC2 activation upregulates VISTA expression. (**A**–**E**) Cohorts of wild-type (WT) mice were intranasally challenged with either rmIL-33 (activated) or PBS (naive). Pulmonary ILC2s were stained for FACS sorting or flow cytometry readout. (**B**–**D**) Pulmonary activated ILC2s (aILC2s) and naive ILC2s (nILC2s) were profiled by scRNA-Seq. (**B**) Left: Uniform manifold approximation and projection (UMAP) of *Vsir*^+^ ILC2s (black dots) isolated from mice receiving PBS (blue panel) or treated with IL-33 (red panel). Right: Violin plot comparing *Vsir* expression level in naive (blue) versus activated (red) ILC2s. (**C**) Heatmap ranking naive (right) and activated (left) ILC2s, based on *Vsir* expression, and exhibiting the transcript levels of Th2/ILC2-related genes from low (green) to high (red). (**D**) Activated ILC2s were stratified into *Vsir^hi^* and *Vsir^lo^*, based on their *Vsir* transcript levels, using the 25% of the population with the lowest expression levels (Q1) as *Vsir^lo^* and the 25% with the highest expression (Q4) as *Vsir^hi^* cells. Dot plot comparing the ILC2-related transcripts in *Vsir^hi^* versus *Vsir^lo^* activated ILC2s. (**E**) Representative plot and bar graph comparing VISTA expression in nILC2s versus aILC2s. Quantification is presented as mean fluorescence intensity (MFI). (**F** and **G**) WT pulmonary nILC2s were treated ex vivo with or without 50 ng/mL rmIL-33. (**G**) Representative plots of VISTA expression levels and corresponding quantification (as MFI). Data are presented as mean + SEM and are representative of at least 2 independent experiments. Statistical significance was assessed using 2-tailed Student’s *t* test (**E**) or 1-way ANOVA followed by Tukey’s post hoc test (**G**); **P < 0.01.

First, we investigated VISTA expression on pulmonary ILC2s. We analyzed the transcriptomic level of the VISTA-encoding gene (*Vsir*) using single-cell RNA sequencing of pulmonary ILC2s isolated from mice challenged with 3 doses of intranasal recombinant mouse IL-33 (rmIL-33) or PBS (Figure 1A) (21). Although a scattered population of naive ILC2s (nILC2s) expressed *Vsir*, a substantial proportion of IL-33–activated ILC2s (aILC2s) showed *Vsir* expression (Figure 1B), indicating inducible VISTA expression at the transcriptome level in aILC2s. We ranked naive and activated ILC2s based on *Vsir* expression, from *Vsir^lo^* to *Vsir^hi^* populations, and compared the transcript levels of Th2/ILC2-related genes in them (Figure 1C). As expected, the expression levels of ILC2 markers were markedly lower in the naive state than in the activated ILC2s. However, in both naive and activated populations, ILC2 activation markers (21) such as *Il5*, *Il6*, *Il9*, *Il13*, *Gata3*, *Csf2*, *Areg*, and *Klrg1* were enriched as the *Vsir* expression decreased (Figure 1C). We next divided activated ILC2s, based on *Vsir* expression, into *Vsir^lo^* and *Vsir^hi^* populations and compared the transcript levels of Th2/ILC2-related genes in these 2 populations (Figure 1D). Notably, ILC2 activation markers as well as *Mki67*, encoding the proliferative marker Ki67, were enriched in *Vsir^lo^* aILC2s (Figure 1D). We also checked the most upregulated genes in *Vsir^hi^* populations and found no Th2/ILC2-related genes in this group (Supplemental Table 1; supplemental material available online with this article; https://doi.org/10.1172/JCI184932DS1). To confirm the inducible expression of VISTA at the protein level, we intranasally administered rmIL-33 and PBS to wild-type (WT) mice for 3 days. Pulmonary ILC2s (live CD45^+^, Lineage^–^, CD127^+^, and ST2^+^ cells) were analyzed via flow cytometry (Supplemental Figure 1, A and B). Although ILC2s showed a basal expression of VISTA in mice receiving PBS, it was significantly upregulated in mice challenged with IL-33 (Figure 1E). We next compared the VISTA expression in ILC2s, eosinophils, and CD4^+^ T cells in our AHR model and found that activated ILC2s expressed higher levels of VISTA protein compared with other subsets (Supplemental Figure 1C). To study the kinetics of VISTA expression on ILC2s following ex vivo activation with rmIL-33, pulmonary nILC2s were cultured ex vivo with or without rmIL-33 for 72 hours (Figure 1F). IL-33 activation progressively increased VISTA expression on aILC2s (Figure 1G). These results suggest that VISTA is inducible at both transcriptome and protein levels in pulmonary ILC2s.

### VISTA deficiency unleashes pulmonary ILC2 effector function.

**Figure 2:**
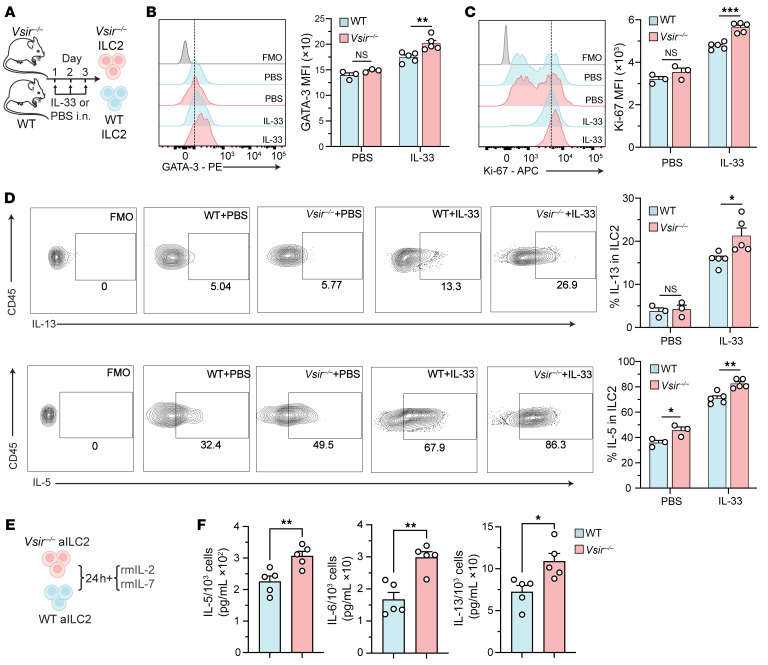
Lack of VISTA enhances pulmonary ILC2 effector function. (**A**–**D**) WT and VISTA-deficient mice were intranasally challenged with rmIL-33 or PBS. Pulmonary ILC2s were stained for flow cytometry readout without ex vivo culture (**B** and **C**) or after stimulation with PMA-ionomycin plus GolgiPlug (**D**). (**B** and **C**) Representative plots of GATA-3 (**B**) and Ki67 (**C**) expression levels and corresponding quantification (as MFI). (**D**) Gating strategy and corresponding quantification comparing the percentage of IL-13^+^ ILC2s and IL-5^+^ ILC2s. (**E** and **F**) Pulmonary aILC2s from WT and VISTA-deficient mice were cultured ex vivo. (**F**) Cytokine levels in cell supernatant (per 10^3^ ILC2s). Data are presented as mean + SEM and are representative of at least 2 independent experiments. Statistical significance was assessed using 2-tailed Student’s *t* test; *P < 0.05, **P < 0.01, ***P < 0.001.

Given the association between VISTA deficiency and multiple inflammatory diseases (9, 11, 17), we investigated the potential role of VISTA in regulating ILC2 effector function, using VISTA-deficient mice. First, we confirmed that VISTA-deficient mice did not express VISTA at the protein level (Supplemental Figure 1D). Next, we compared the number of ILC2s, activation and proliferation markers, cytokine levels, and lung histology between VISTA-deficient and WT mice at steady state. Both groups exhibited comparable ILC2 numbers and similar levels of ILC2 activation and proliferation, with no differences in lung epithelial thickness (Supplemental Figure 2, A–G). Cohorts of WT and VISTA-deficient mice were intranasally challenged with rmIL-33 or PBS for 3 days (Figure 2A). Freshly isolated pulmonary ILC2s were then analyzed for GATA-3, the key transcription factor regulating ILC2 maintenance and function, Ki67, a marker of cell proliferation, and intracellular IL-5 and IL-13 as ILC2 effector cytokines. While WT and *Vsir^–/–^* ILC2s exhibited comparable GATA-3 and Ki67 expression at steady state, we observed a significant increase in both GATA-3 and Ki67 across in vivo–activated *Vsir^–/–^* ILC2s, compared with their WT counterparts (Figure 2, B and C). The frequency of IL-13^+^ nILC2s was similar between WT and VISTA-deficient mice; however, the percentage of IL-13^+^ aILC2s was significantly higher in mice lacking VISTA compared with WT controls (Figure 2D). Interestingly, the frequency of IL-5^+^ ILC2s was also remarkably elevated in VISTA-deficient mice compared with WT controls (Figure 2D). To assess the role of VISTA in the cytokine production capacity of ILC2s, pure populations of pulmonary aILC2s were FACS-sorted from WT and VISTA-deficient mice and cultured ex vivo with rmIL-2 and rmIL-7 for 24 hours (Figure 2E). We found that *Vsir^–/–^* ILC2s secreted significantly higher levels of IL-5, IL-6, and IL-13 compared with WT ILC2s in the culture supernatants (Figure 2F). To confirm the findings from VISTA-deficient mice, we blocked VISTA on WT ILC2s using a well-documented VISTA antagonist antibody (13F3) (19). Preliminary apoptosis assays evaluating 13F3 toxicity showed no harmful effects on ILC2s (Supplemental Figure 2H). Interestingly, blocking VISTA in WT ILC2s elevated the GATA-3 and Ki67 levels, and increased the secretion of type 2 cytokines (Supplemental Figure 2, I–L). Collectively, these findings demonstrate that deficiency or blocking of VISTA unleashes ILC2 effector function ex vivo, underscoring its critical role in ILC2 regulation.

### Lack of VISTA exacerbates ILC2-mediated AHR and lung inflammation.

**Figure 3:**
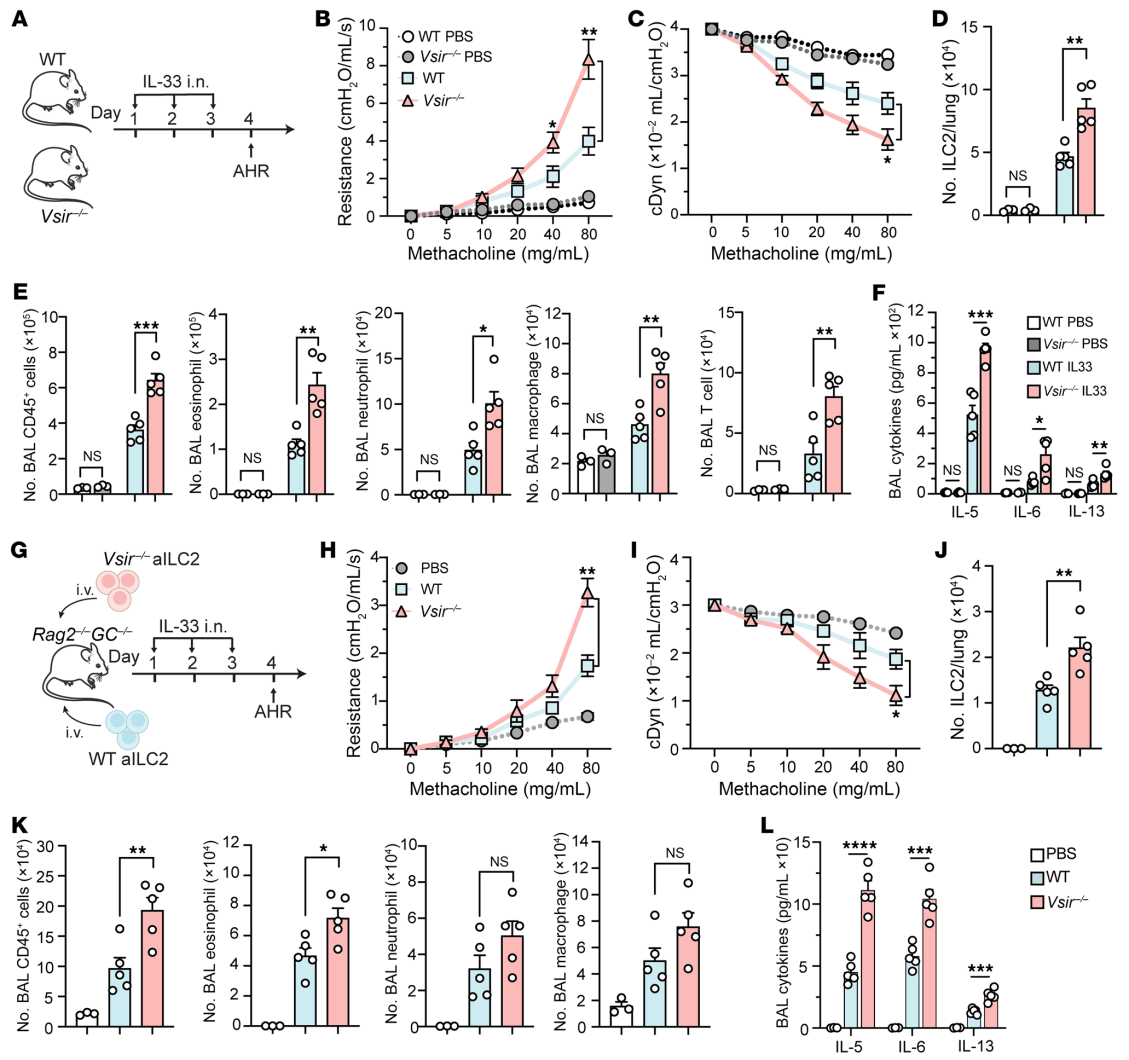
The absence of VISTA aggravates ILC2-driven AHR and lung inflammation. (**A**–**F**) WT and VISTA-deficient mice were intranasally challenged with rmIL-33 or PBS. (**B** and **C**) Lung resistance (**B**) and dynamic compliance (**C**) in response to elevating doses of methacholine. (**D**) Total number of ILC2s per lung. (**E**) Total number of CD45^+^ cells, eosinophils, neutrophils, macrophages, and T cells in BAL fluid. (**F**) Cytokine levels in the BAL fluid. (**G**–**L**) Cohorts of *Rag^–/–^GC^–/–^* mice were intravenously injected with WT or *Vsir^–/–^* aILC2s and intranasally challenged with rmIL-33 or PBS. (**H** and **I**) Lung resistance (**H**) and dynamic compliance (**I**) in response to elevating doses of methacholine. (**J**) Total number of ILC2s per lung. (**K**) Total number of CD45^+^ cells, eosinophils, neutrophils, and macrophages in BAL fluid. (**L**) Cytokine levels in the BAL fluid. Data are presented as mean + SEM and are representative of at least 2 independent experiments. Statistical significance was assessed using 1-way ANOVA followed by Tukey’s post hoc test; *P < 0.05, **P < 0.01, ***P < 0.001, ****P < 0.0001.

Given the pivotal role of ILC2s in the development and progression of AHR and pulmonary inflammation (3, 4), we next evaluated the impact of VISTA deficiency on IL-33–induced AHR and lung inflammation. WT and VISTA-deficient mice were challenged with intranasal rmIL-33 for 3 days, followed by lung function testing with methacholine on day 4. After euthanasia, bronchoalveolar lavage (BAL) samples and lungs were collected for flow cytometry and cytokine assays (Figure 3A). We observed a remarkably increased level of lung resistance in mice given IL-33 compared with PBS controls, thereby validating the IL-33–induced AHR model. Notably, mice lacking VISTA exhibited significantly higher lung resistance than WT mice (Figure 3B). Assessment of dynamic compliance revealed reduced elasticity in the lung of mice lacking VISTA compared with WT controls (Figure 3C). Furthermore, the total number of recovered ILC2s from mice lacking VISTA significantly increased in comparison with WT mice (Figure 3D). Analysis of immune cells in BAL samples, as depicted in Supplemental Figure 3A, showed a substantial increase in total CD45^+^ cells — in particular, eosinophils, neutrophils, macrophages, and T cells — in mice lacking VISTA, indicating increased inflammation due to VISTA deficiency (Figure 3E). Macrophages in the BAL fluid were Gr-1^–^, CD11c^+^, CD64^+^, CD88^+^, CD206^+^, CD11b^lo/–^, and MHC-II^lo/–^, consistent with previously reported phenotypic profiles (Supplemental Figure 3A) (22). Interestingly, the activation, proliferation, and function of Th2 cells were not different across WT and VISTA-deficient mice following intranasal IL-33 challenge (Supplemental Figure 3, B–D). Consistent with higher ILC2 numbers in VISTA-deficient mice, elevated levels of IL-5, IL-6, and IL-13 were detected in BAL samples from VISTA-deficient mice compared with WT counterparts (Figure 3F). Histological examination of lung tissue supported these observations, demonstrating increased airway epithelium thickness in VISTA-deficient mice following IL-33 activation compared with WT mice (Supplemental Figure 4, A and B). These observations suggest that VISTA deficiency leads to more severe AHR and lung inflammation. Since VISTA is expressed on various immune cells, we investigated whether deficiency specifically on ILC2s could influence AHR and lung inflammation. To address this, we adoptively transferred WT and *Vsir^–/–^* ILC2s into cohorts of *Rag2^–/–^GC^–/–^* alymphoid mice. Mice were then challenged intranasally with rmIL-33 (Figure 3G). As anticipated, adoptive transfer of WT ILC2s induced AHR, as evidenced by higher lung resistance and lower dynamic compliance, in comparison with non-transferred mice (Figure 3, H and I). Interestingly, however, mice adoptively transferred with *Vsir^–/–^* ILC2s exhibited significantly higher lung resistance and lower dynamic compliance compared with the group receiving WT ILC2s (Figure 3, H and I). The exacerbated lung inflammation in mice with transferred *Vsir^–/–^* ILC2s was evidenced by increased ILC2 numbers in the lung (Figure 3J) with higher Ki67 levels (Supplemental Figure 4C), elevated CD45^+^ cells and eosinophil counts in BAL (Figure 3K), higher levels of IL-5, IL-6, and IL-13 in BAL (Figure 3L), and thicker airway epithelium (Supplemental Figure 4, D and E), in comparison with the control group. To evaluate whether blocking VISTA has the same effect as VISTA deficiency on ILC2-mediated AHR, WT mice were intranasally challenged with rmIL-33 and treated intraperitoneally with anti–VISTA antagonist antibody. Mice receiving the antagonist exhibited significantly higher lung resistance, increased pulmonary ILC2 numbers, and elevated CD45^+^ cells and eosinophils in BAL compared with controls (Supplemental Figure 4, F and G). Additionally, BAL samples from these mice showed increased levels of IL-5, IL-6, and IL-13 compared with controls (Supplemental Figure 4H). These findings underscore that VISTA deficiency or blocking in ILC2 exacerbates ILC2-dependent AHR and airway inflammation.

### VISTA modulates ILC2 activity through the FOXO1 pathway.

**Figure 4:**
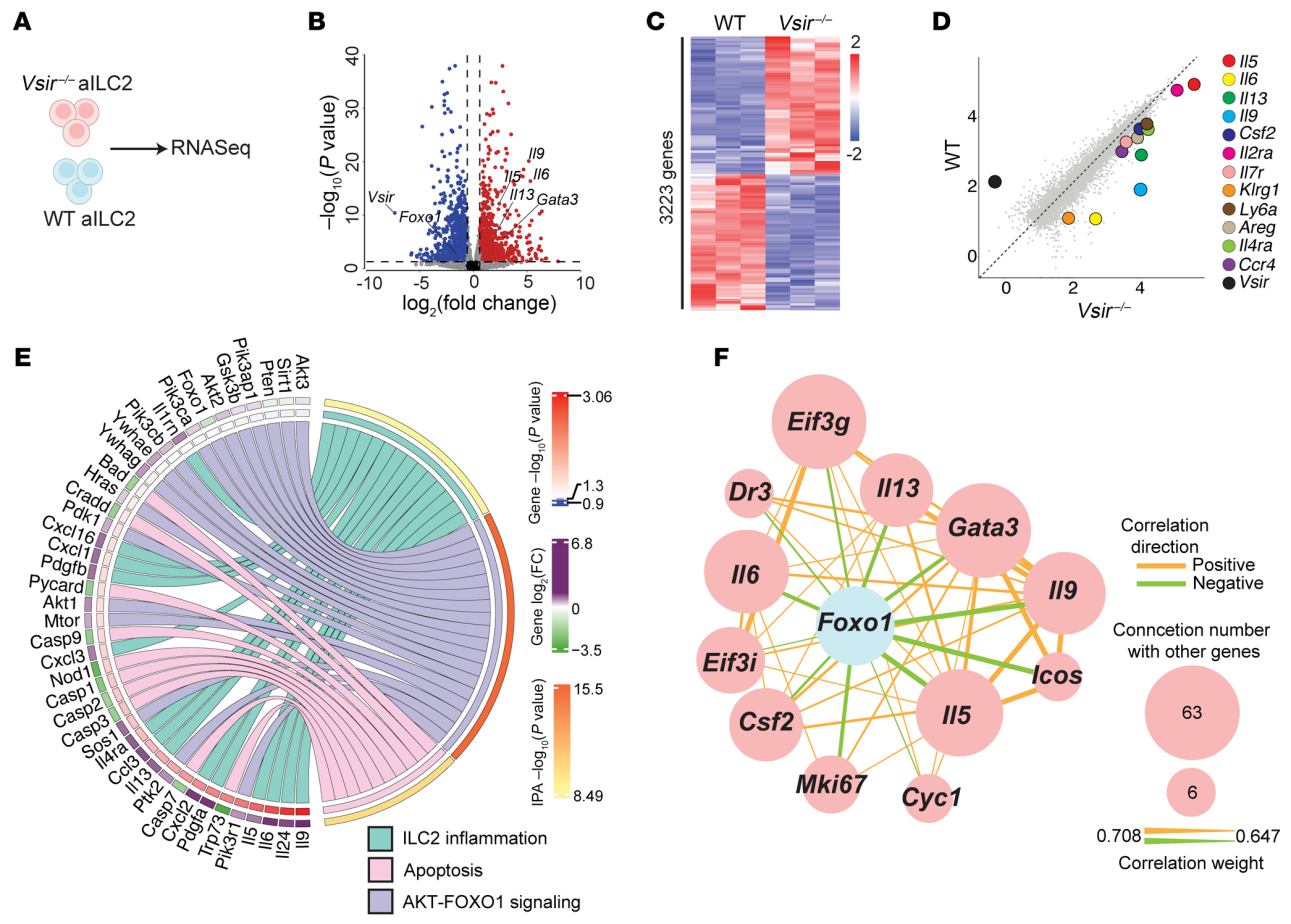
AKT/FOXO1 pathway is affected in VISTA-deficient ILC2s. (**A**–**F**) WT and VISTA-deficient mice received 3 days of intranasal rmIL-33. Total RNA was extracted from isolated pulmonary aILC2s to perform a bulk transcriptomic analysis. (**B**) Volcano plot comparison between WT and *Vsir^–/–^* aILC2s. (**C**) Heatmap of differentially modulated transcripts. (**D**) Dot plot of the differentially modulated ILC2-related transcripts. (**E**) Chord plot representing the most highly differentially expressed genes from top upregulated pathways. Specific pathways are color-coded and represented in the right inner bands, where chords gather. Outer bands (yellow to red) on the right depict the Ingenuity Pathway Analysis (IPA) –log_10_ *P* value. The left inner bands (blue to red) represent the gene –log_10_ *P* value. The left outer bands (green to purple) represent the gene log_2_(fold change). (**F**) Network visualization of weighted correlation network analysis for selected genes in the most correlated module, with correlations larger than the mean overall correlation score within that module. Orange lines represent positive correlations and green lines represent negative correlations between genes within the module. The size of each node corresponds to the number of connections one gene has with other genes within the module. The thickness of the edges indicates the strength of the correlation.

Having established the regulatory effects of VISTA on pulmonary ILC2s and ILC2-mediated AHR, we investigated the mechanism by which VISTA influences ILC2 function. To address this, we conducted a transcriptomic analysis using pure populations of activated ILC2s isolated from WT and VISTA-deficient mice (Figure 4A). As depicted in Figure 4, B and C, we identified 3,223 differentially expressed genes, confirming a distinct transcriptional signature induced by VISTA deficiency. We found that transcripts associated with ILC2/Th2 responses were substantially upregulated in *Vsir^–/–^* ILC2s (Figure 4D). Pathway enrichment analysis further showed that VISTA deficiency resulted it hyperactivation in ILC2s while dampening apoptosis-related gene expression in comparison with WT controls (Figure 4E). To confirm the transcriptome findings on apoptosis-related genes, we assessed the protein levels of caspase-3/7, phosphorylated BAD (p-BAD), and BCL2, and found that the frequency of caspase-3/7^+^ cells and the levels of p-BAD were lower in *Vsir^–/–^* ILC2s compared with their WT counterparts (Supplemental Figure 5, A and B). Furthermore, *Vsir^–/–^* ILC2s exhibited higher expression levels of BCL2 in comparison with WT controls (Supplemental Figure 5C). Together, these results confirm our transcriptome findings that apoptosis is downregulated in the absence of VISTA.

**Figure 5:**
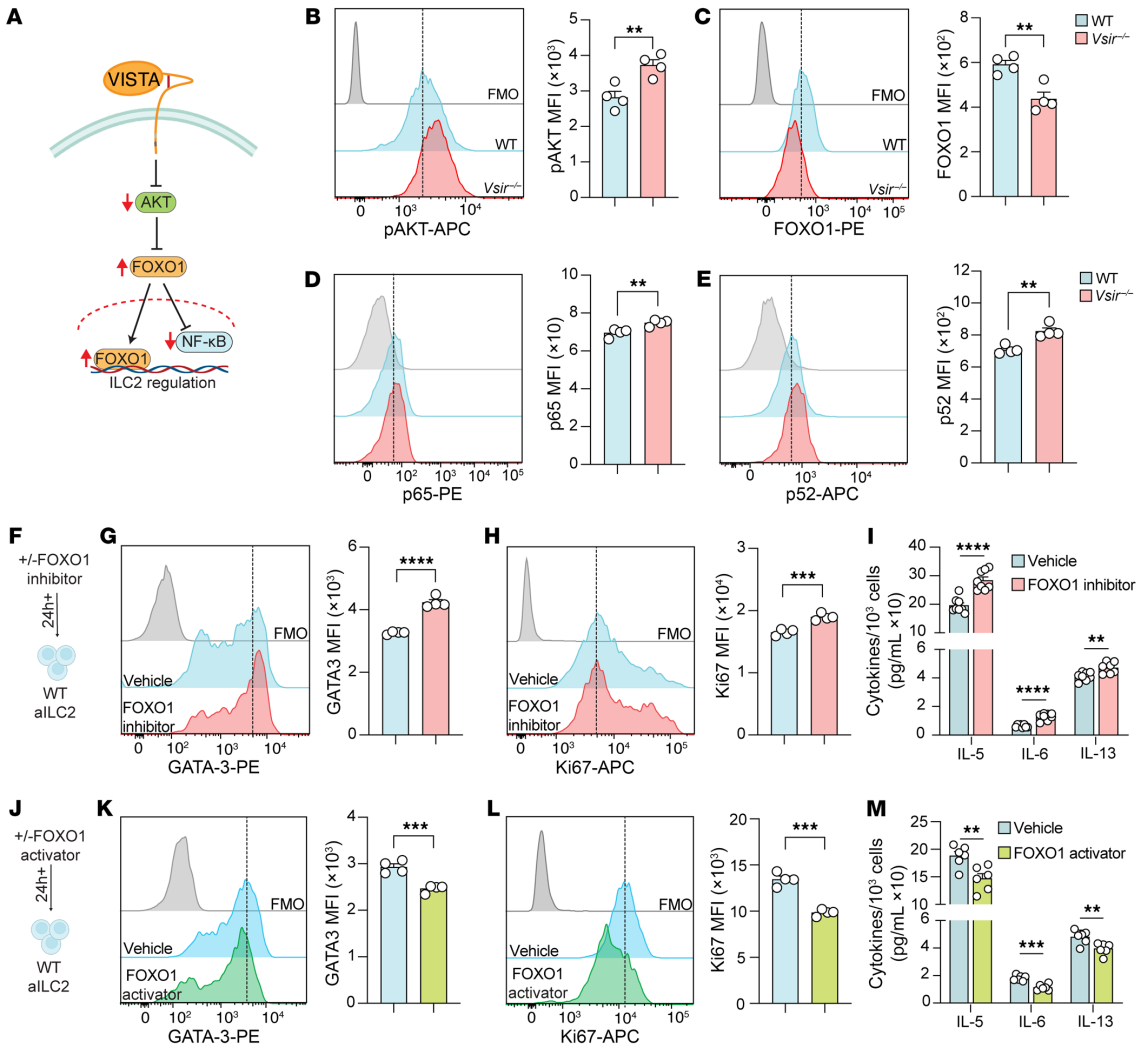
FOXO1 pathway activation by VISTA regulates ILC2 function. (**A**) Overview of VISTA downstream signaling. (**B**–**E**) Freshly isolated WT and *Vsir^–/–^* aILC2s were stained intranuclearly for p-AKT, FOXO1, p65, and p52. Representative histograms of protein expression of p-AKT (**B**), FOXO1 (**C**), p65 (**D**), and p52 (**E**), and corresponding quantifications as MFI. (**F**–**I**) WT aILC2s were treated with FOXO1 inhibitor or vehicle. (**G** and **H**) Representative plots of GATA-3 (**G**) and Ki67 (**H**) expression levels and corresponding quantification (as MFI). (**I**) Cytokine levels in cell supernatant (per 10^3^ ILC2s). (**J**–**M**) WT aILC2s were treated with FOXO1 activator or vehicle. (**K** and **L**) Representative plots of GATA-3 (**K**) and Ki67 (**L**) expression levels and corresponding quantification (as MFI). (**M**) Cytokine levels in cell supernatant (per 10^3^ ILC2s). Data are presented as mean + SEM and are representative of at least 2 independent experiments. Statistical significance was assessed using 2-tailed Student’s *t* test; **P < 0.01, ***P < 0.001, ****P < 0.0001.

In pursuit of the molecular mechanism behind the ILC2 hyperactivation, we found that the AKT/FOXO1 pathway was remarkably upregulated by the lack of VISTA (Figure 4E). Previous studies have established that AKT inhibits FOXO1 by promoting its degradation (23, 24). Accordingly, we found upregulation in *Akt* isoforms and other AKT signaling molecules (Figure 4E). On the other hand, we observed a downregulation in *Foxo1* and its activating molecules, such as *Pten* and *Sirt1* (Figure 4E) (23). To further explore the correlation between *Foxo1* and ILC2 activation, we used weighted gene coexpression network analysis (WGCNA). Intriguingly, we found significant negative correlations between *Foxo1* and several markers linked to ILC2 activation and proliferation, including *Gata3*, *Il5*, *Il13*, *Il9*, *Il6*, *Csf2*, *Icos*, and *Mki67* (Figure 4F). Based on these insights, we hypothesized that VISTA might exert its regulatory effects on ILC2s through the AKT/FOXO1 pathway (Figure 5A). To test our hypothesis, we examined the phosphorylation of AKT (p-AKT) and FOXO1 protein level in freshly isolated WT and *Vsir^–/–^* ILC2s. VISTA deficiency led to increased p-AKT (Figure 5B) and decreased FOXO1 levels (Figure 5C). To confirm that these effects are mediated by VISTA signaling, we treated WT ILC2s with well-documented anti–VISTA agonist and anti–VISTA antagonist antibodies (MH5A and 13F3, respectively) (17, 19, 20, 25). Preliminary apoptosis assays assessing MH5A toxicity revealed no harmful effects on ILC2s (Supplemental Figure 5D). Consistent with findings in *Vsir^–/–^* ILC2s, blocking VISTA on WT ILC2s elevated p-AKT levels and reduced FOXO1 protein levels (Supplemental Figure 5, E–G). Conversely, VISTA agonist treatment resulted in decreased p-AKT levels and elevated FOXO1 protein levels (Supplemental Figure 5, F and G). These observations indicate that VISTA signaling leads to FOXO1 upregulation in ILC2s, suggesting that VISTA effects on ILC2s might be at least in part through the FOXO1 pathway. To further characterize the downstream effects of the AKT/FOXO1 pathway in ILC2s, we examined the phosphorylation of p65 and p52 proteins, major subunits of canonical and non-canonical NF-κB, respectively, in freshly isolated WT and *Vsir^–/–^* ILC2s. Notably, VISTA deficiency led to increased levels of both p65 and p52 (Figure 5, D and E), suggesting that the role of VISTA via FOXO1 signaling may be linked to both canonical and non-canonical NF-κB signaling. Next, we questioned whether FOXO1 affects ILC2 proliferation and/or effector function. To address this, WT aILC2s were treated with AS1842856, a well-documented FOXO1 inhibitor (26), or vehicle control (Figure 5F). Preliminary assessment of AS1842856 toxicity using apoptosis assays showed no adverse effects on ILC2s (Supplemental Figure 5H). Strikingly, FOXO1 inhibition increased the expression of GATA-3 and Ki67 in comparison with the vehicle control (Figure 5, G and H). In addition, the levels of IL-5, IL-6, and IL-13 in the culture supernatant were elevated following FOXO1 inhibition in ILC2s (Figure 5I). To further confirm the regulatory role of FOXO1 in ILC2s, we used LOM612, known to activate FOXO1 signaling by relocating it to the nucleus (26, 27). WT aILC2s were treated with either LOM612 or vehicle control (Figure 5J). As reported for AS1842856, apoptosis assay showed no toxic effects following treatment of ILC2s with LOM612 (Supplemental Figure 5I). Interestingly, however, FOXO1 activation resulted in downregulation of both GATA-3 and Ki67 in ILC2s compared with vehicle treatment (Figure 5, K and L), accompanied by reduced secretion of IL-5, IL-6, and IL-13 from ILC2s (Figure 5M). In summary, our findings demonstrate that VISTA signaling upregulates FOXO1 and inhibits NF-κB signaling, which in turn suppresses ILC2 effector function.

### VISTA-deficient ILC2s exhibit enhanced metabolic activity.

**Figure 6:**
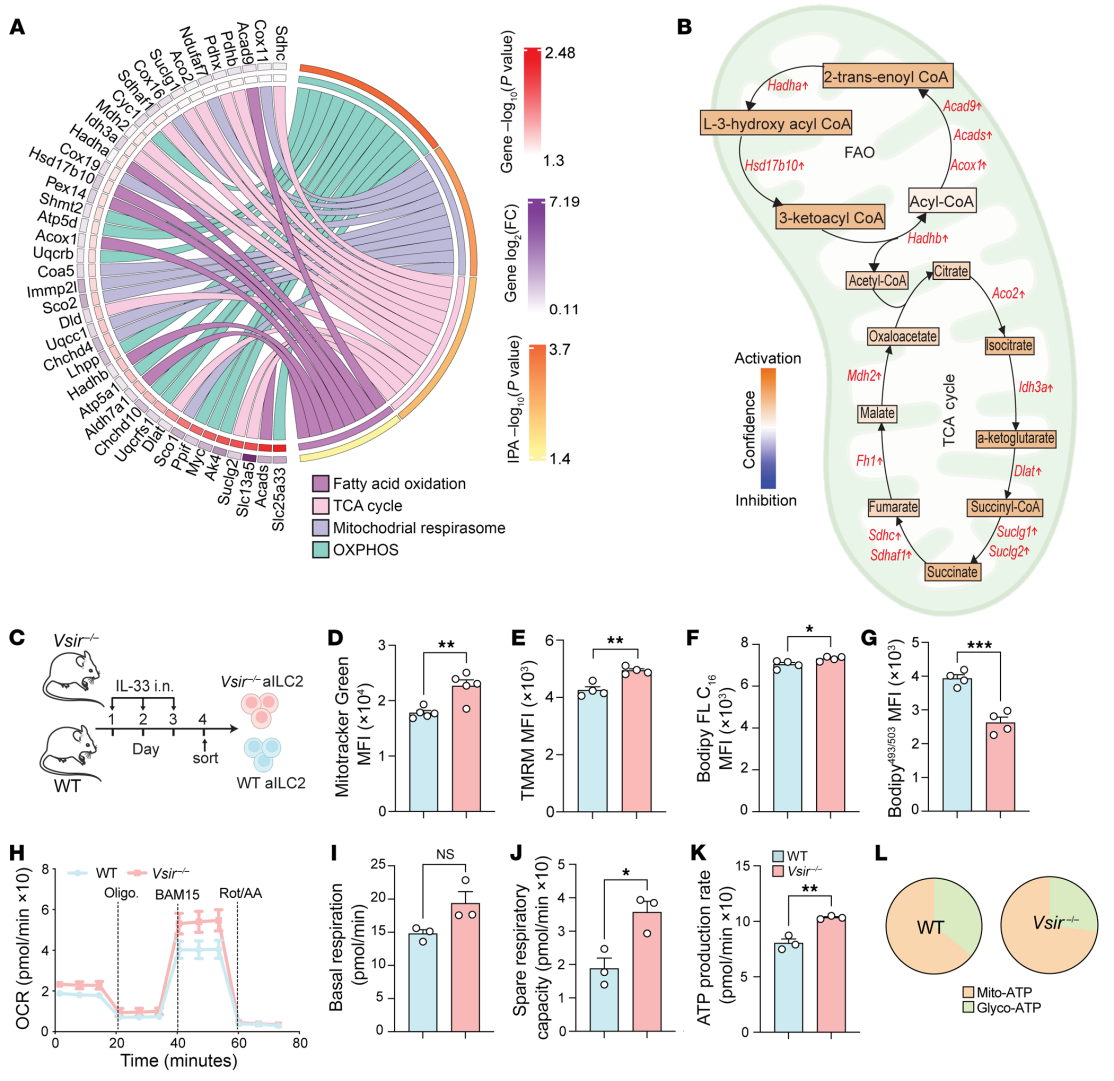
ILC2s lacking VISTA exhibit higher mitochondrial respiration and FAO. (**A** and **B**) WT and VISTA-deficient mice received 3 days of intranasal rmIL-33. Total RNA was extracted from isolated pulmonary aILC2s to perform a bulk transcriptomic analysis. (**A**) Chord plot representing the most highly differentially expressed genes from top upregulated metabolic pathways. Specific pathways are color-coded and represented in the right inner bands, where chords gather. Outer bands (yellow to red) on the right depict the IPA –log_10_ *P* value. The left inner bands (white to red) represent the gene –log_10_ *P* value. The left outer bands (white to purple) represent the gene log_2_(fold change). (**B**) Schematic of TCA cycle and FAO showing the trends of enzymes differentially modulated in *Vsir^–/–^* versus WT aILC2s, with the color-coding trends of intermediate metabolites based on IPA analysis (blue to orange for inhibition to activation). (**C**–**L**) WT and *Vsir^–/–^* aILC2s underwent metabolic analyses. (**D**) Bar plots showing the quantification of MitoTracker Green (as MFI). (**E**) Bar plots showing the quantification of TMRM in freshly isolated ILC2s (as MFI). (**F** and **G**) BODIPY FL C_16_ (**F**) and BODIPY^493/503^ (**G**) expression levels (as MFI). (**H**–**L**) Mitochondrial respiratory profile. (**H**) Oxygen consumption rate (OCR) in response to oligomycin, BAM15, and rotenone plus antimycin A (Rot/AA) sequential injections. (**I**–**L**) Basal respiration (**I**), spare respiratory capacity (**J**), ATP production rate (**K**), and mitochondrial ATP (mito-ATP) and glycolytic ATP (glycol-ATP) production (**L**). Data are presented as mean + SEM and are representative of at least 2 independent experiments. Statistical significance was assessed using 2-tailed Student’s *t* test; *P < 0.05, **P < 0.01, ***P < 0.001.

Previous studies have demonstrated that FOXO1 activation suppresses cellular metabolism, including FAO and OXPHOS in both immune and non-immune cells (28, 29). Since activated ILC2s mainly use FAO and OXPHOS to meet energy demands, we investigated whether VISTA-mediated regulation of FOXO1 impacts ILC2 metabolism. Pathway analysis of transcriptome data from *Vsir^–/–^* and WT aILC2s revealed that OXPHOS, mitochondrial respirasome, the TCA cycle, and FAO were among the top enriched metabolic pathways, all of which were upregulated in *Vsir^–/–^* ILC2s (Figure 6A). The schematic of FAO and the TCA cycle in Figure 6B illustrates the location and function of enzymes with transcripts listed in Figure 6A. According to the results of Ingenuity Pathway Analysis, higher expression of enzymes involved in FAO can lead to elevated acetyl-CoA production, fueling the TCA cycle to meet the energy demands of ILC2s (Figure 6B). This finding indicates that at the transcriptome level, VISTA deficiency enhances the metabolic pathways primarily used by activated ILC2s for proliferation and effector function. To validate the transcriptomic results, we performed metabolic analyses on pure populations of WT and *Vsir^–/–^* aILC2s (Figure 6C). We first found that *Vsir^–/–^* ILC2s exhibited significantly higher MitoTracker Green staining, suggesting increased mitochondrial mass compared with WT controls (Figure 6D). Additionally, we measured the mitochondrial membrane potential using tetramethylrhodamine methyl ester (TMRM), a widely used fluorescent dye for assessing mitochondrial membrane potential in live cells (30). Our findings indicated that *Vsir^–/–^* ILC2s exhibited higher TMRM intensity, suggesting an elevated mitochondrial membrane potential (Figure 6E). To evaluate FAO status, BODIPY FL C_16_ and BODIPY^493/503^ were used to measure fatty acid uptake and fatty acid accumulation, respectively. VISTA deficiency was associated with increased fatty acid uptake (Figure 6F) and decreased fatty acid accumulation (Figure 6G), suggesting higher FAO activity in *Vsir^–/–^* ILC2s. We further assessed the impact of VISTA deficiency on mitochondrial respiration in ILC2s using a bioenergetic assay (Figure 6H). Consistent with our transcriptomic analysis, *Vsir^–/–^* activated ILC2s demonstrated higher basal respiration (Figure 6I), spare respiratory capacity (Figure 6J), and ATP production rates (Figure 6K), indicating enhanced oxidative metabolism. Furthermore, mitochondria-mediated ATP production was greater in *Vsir^–/–^* ILC2s compared with WT ILC2s (Figure 6L). Overall, the combined transcriptomic, metabolic, and bioenergetic analyses indicate that VISTA deficiency enhances metabolic pathways in ILC2s, facilitating their proliferation and effector function. Next, we asked whether VISTA ligation on ILC2s can affect the ILC2 metabolic activity (Supplemental Figure 6A). Strikingly, ligation of VISTA on WT ILC2s reduced mitochondrial mass and membrane potential, as evidenced by lower MitoTracker Green and TMRM (Supplemental Figure 6, B and C). Consistently, bioenergetic assay (Supplemental Figure 6D) exhibited lower levels of basal respiration, spare respiratory capacity, ATP production rate, and mitochondrial ATP production (Supplemental Figure 6, E–H) following treatment with VISTA agonist. These findings suggest that VISTA ligation reduces metabolic activity in ILC2s.

### VISTA engagement restrains ILC2 activation and ILC2-mediated AHR development.

**Figure 7:**
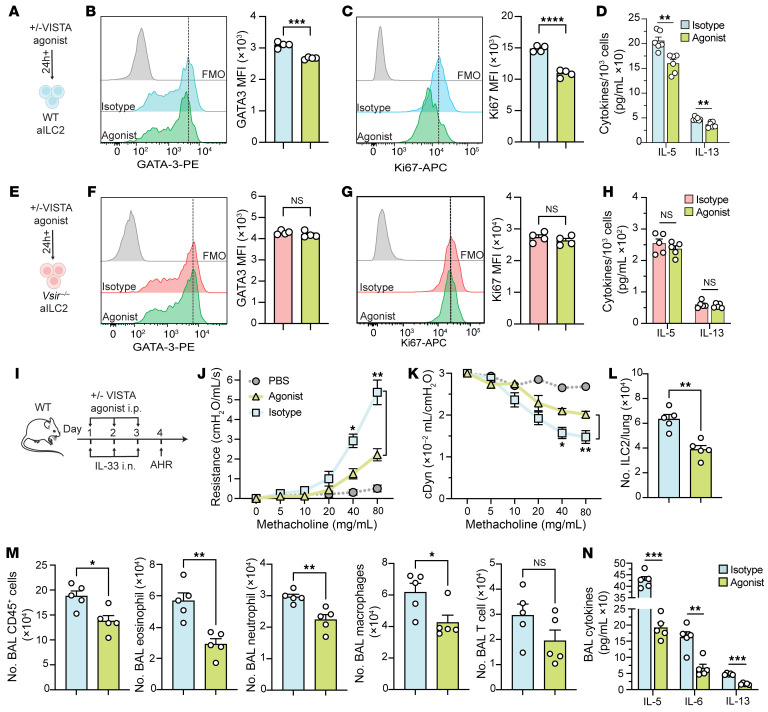
VISTA agonist reduces ILC2 function and alleviates AHR. (**A**–**D**) WT aILC2s were treated with VISTA agonist antibody or isotype control. (**B** and **C**) Representative plots of GATA-3 (**B**) and Ki67 (**C**) expression levels and corresponding quantification (as MFI). (**D**) Cytokine levels in cell supernatant (per 10^3^ ILC2s). (**E**–**H**) *Vsir^–/–^* aILC2s were treated with VISTA agonist antibody or isotype control. (**F** and **G**) Representative plots of GATA-3 (**F**) and Ki67 (**G**) expression levels and corresponding quantification (as MFI). (**H**) Cytokine levels in cell supernatant (per 10^3^ ILC2s). (**I**–**N**) WT mice were intraperitoneally injected with 5 mg/kg of anti–mouse VISTA agonist antibody or isotype control, followed by intranasal challenge with rmIL-33 or PBS for 3 days. (**J** and **K**) Lung resistance (**J**) and dynamic compliance (**K**) in response to elevating doses of methacholine. (**L**) Total number of ILC2s per lung. (**M**) Total number of CD45^+^ cells, eosinophils, neutrophils, macrophages, and T cells in BAL fluid. (**N**) Cytokine levels in the BAL fluid. Data are presented as mean + SEM and are representative of at least 2 independent experiments. Statistical significance was assessed using either 2-tailed Student’s *t* test (**B**–**H** and **L**–**N**) or 1-way ANOVA followed by Tukey’s post hoc test (**J** and **K**); *P < 0.05, **P < 0.01, ***P < 0.001, ****P < 0.0001.

So far, we have confirmed the role of VISTA in regulating ILC2 function and ILC2-mediated airway inflammation. To evaluate VISTA as a potential therapeutic target for restraining ILC2-driven AHR, we designed sets of experiments using a VISTA agonist antibody. WT aILC2s were treated with a VISTA agonist or isotype control for 24 hours (Figure 7A). We found that VISTA agonist significantly reduced the levels of GATA-3 and Ki67 (Figure 7, B and C). Additionally, VISTA engagement on ILC2s decreased the secretion of IL-5 and IL-13 (Figure 7D). These results suggest that VISTA engagement ex vivo restrains ILC2 proliferation and effector function. To ensure that the effects of the agonist antibody were VISTA specific, we treated *Vsir^–/–^* ILC2s with the anti–VISTA agonist antibody (Figure 7E) and observed no significant changes in GATA-3, Ki67, or cytokine levels (Figure 7, F–H) between the agonist-treated and isotype control groups. This confirms that the agonist antibody regulates ILC2 functions in a VISTA-dependent manner. Next, we questioned whether VISTA suppresses only IL-33–mediated activation of ILC2s. We used IL-25, TSLP, and IL-33 as key alarmins that activate ILC2s, and our results indicate that VISTA expression was most strongly induced following activation with IL-33 (Supplemental Figure 6I). While we also observed a significant increase in VISTA induction with IL-25, activation of ILC2s with TSLP in vivo did not modulate VISTA expression (Supplemental Figure 6I). Therefore, we investigated whether a VISTA agonist could suppress IL-25–mediated activation of ILC2s. Consistent with our findings using IL-33 as an ILC2 activator, we observed that the VISTA agonist also decreased GATA-3 and Ki67 expression in IL-25–activated ILC2s (Supplemental Figure 6, J and K). Furthermore, the levels of secreted IL-5 and IL-13 were significantly lower in the VISTA agonist–treated group compared with the control group (Supplemental Figure 6L). Our findings confirm that the regulatory effects of VISTA on ILC2s are not merely limited to IL-33 activation.

Building on the ex vivo effect of VISTA engagement in ILC2 regulation, we next evaluated whether in vivo administration of the VISTA agonist could reduce the development of AHR and lung inflammation. Cohorts of WT mice were intranasally challenged with rmIL-33 and received either a VISTA agonist or an isotype control intraperitoneally before each intranasal challenge. On day 4, mice underwent pulmonary function tests, BAL examinations, and histological analysis (Figure 7I). Mice challenged with IL-33 rather than PBS exhibited elevated lung resistance, validating the IL-33–induced AHR model. Notably, results showed a significant reduction in lung resistance and improved dynamic compliance in the VISTA agonist–treated group compared with the isotype control group (Figure 7, J and K). These improvements were coupled with a decrease in pulmonary ILC2s (Figure 7L), a reduced number of BAL CD45^+^ cells, eosinophils, neutrophils, and macrophages (Figure 7M), and lower levels of BAL IL-5, IL-6, and IL-13 (Figure 7N) in the VISTA agonist–treated group compared with controls. Histological examination revealed that mice receiving the VISTA agonist exhibited reduced airway epithelium thickness compared with the isotype control group (Supplemental Figure 7, A and B). These findings suggest that VISTA engagement suppresses ILC2 function and alleviates ILC2-mediated airway hyperreactivity and inflammation.

### Treatment with a VISTA agonist alleviates ILC2-driven AHR in an IL-33–induced model.

**Figure 8:**
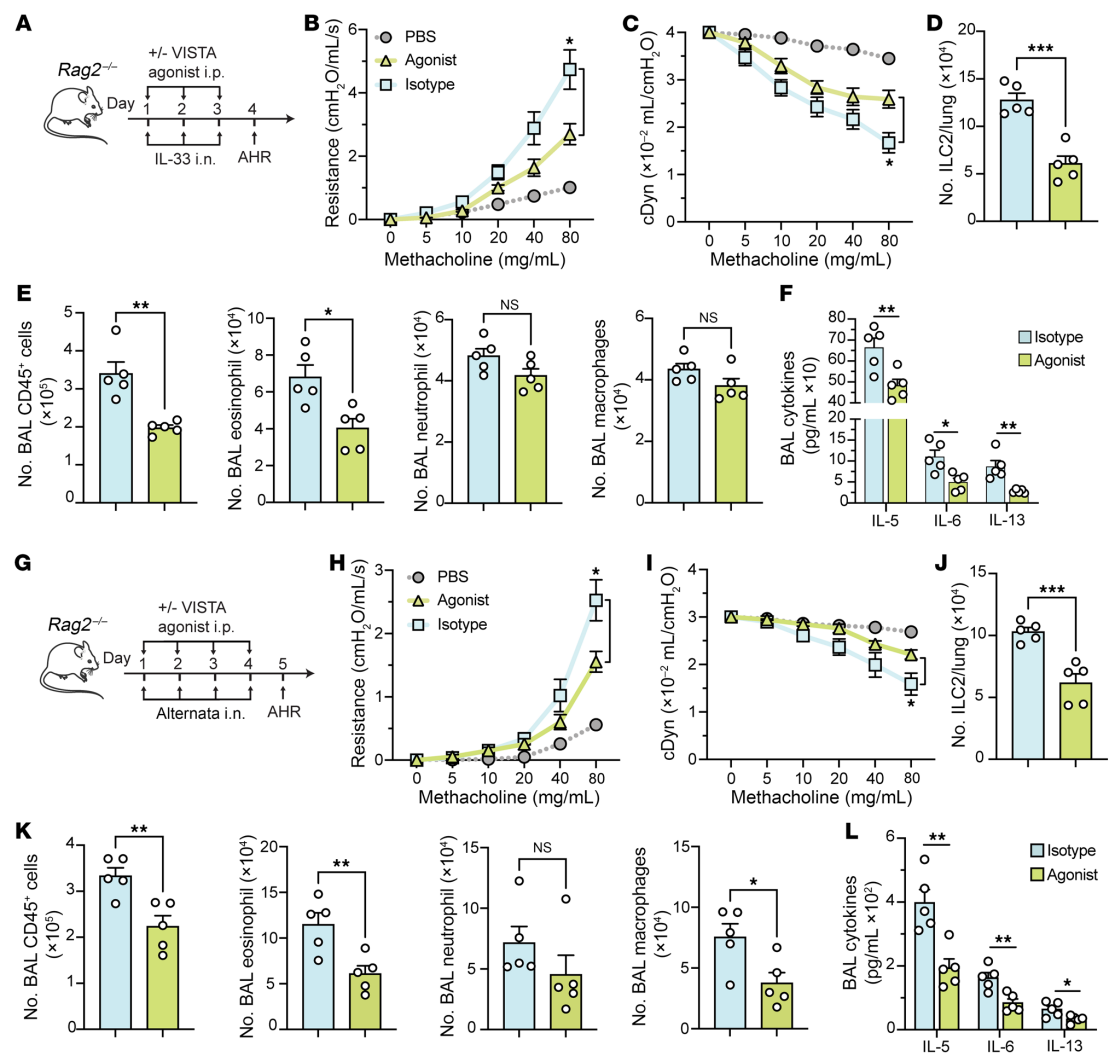
Treatment with VISTA agonist limits ILC2-driven AHR in both IL-33– and allergen-induced models. (**A**–**F**) *Rag2^–/–^* mice were intraperitoneally injected with 5 mg/kg of anti–VISTA agonist antibody or isotype control, followed by intranasal challenge with rmIL-33 or PBS. (**B** and **C**) Lung resistance (**B**) and dynamic compliance (**C**) in response to elevating doses of methacholine. (**D**) Total number of ILC2s per lung. (**E**) Total number of CD45^+^ cells, eosinophils, neutrophils, and macrophages in BAL fluid. (**F**) Cytokine levels in the BAL fluid. (**G**–**L**) *Rag2^–/–^* mice were intraperitoneally injected with 5 mg/kg of anti–VISTA agonist antibody or isotype control, followed by intranasal challenge with *A*. *alternata* or PBS. (**H** and **I**) Lung resistance (**H**) and dynamic compliance (**I**) in response to elevating doses of methacholine. (**J**) Total number of ILC2s per lung. (**K**) Total number of CD45^+^ cells, eosinophils, neutrophils, and macrophages in BAL fluid. (**L**) Cytokine levels in the BAL fluid. Data are presented as mean + SEM and are representative of at least 2 independent experiments. Statistical significance was assessed using either 2-tailed Student’s *t* test (**D**–**F** and **J**–**L**) or 1-way ANOVA followed by Tukey’s post hoc test (**B**, **C**, **H**, and **I**); *P < 0.05, **P < 0.01, ***P < 0.001.

Th2 cells and ILC2s are recognized as major contributors to the development of allergic AHR (4). Therefore, we investigated whether a VISTA agonist could ameliorate AHR in the absence of Th2 cells. We induced IL-33–mediated AHR in *Rag2*-deficient (*Rag2^–/–^*) mice and administered a VISTA agonist antibody or isotype control (Figure 8A). First, we found a significant increase in lung resistance and a remarkable decrease in dynamic compliance in mice receiving IL-33 rather than PBS, validating the IL-33–induced AHR. In mice challenged with rmIL-33, the VISTA agonist significantly reduced lung resistance and improved dynamic compliance (Figure 8, B and C). The total number of ILC2s in the lungs was significantly decreased in the group treated with the VISTA agonist compared with control (Figure 8D). Additionally, VISTA engagement led to lower lung inflammation, as evidenced by reduced numbers of CD45^+^ cells and eosinophils in the BAL (Figure 8E). Decreased levels of IL-5, IL-6, and IL-13 were also observed in mice treated with a VISTA agonist compared with control mice (Figure 8F). Histological analysis supported these results, showing that VISTA engagement reduced the thickening of the airway epithelium (Supplemental Figure 7, C and D). These results elucidate that VISTA engagement limits the development of ILC2-dependent AHR and lung inflammation.

### Treatment with a VISTA agonist alleviates ILC2-driven AHR in an allergen-induced model.

To further explore the impact of a VISTA agonist on ILC2-mediated AHR in response to a clinically relevant allergen, we used *Alternaria alternata*–induced AHR in *Rag2*-deficient mice. *A*. *alternata* is a prevalent mold in environmental samples, recognized for its potent allergenic properties. Sensitization to this mold has been linked to increased asthma severity (31). Importantly, it has been recognized as a primary allergen associated with asthma in American households (31). *Rag2^–/–^* mice received intraperitoneal injections of a VISTA agonist or an isotype control and were intranasally exposed to *A*. *alternata* over 4 days. On day 5, pulmonary function was tested, lung ILC2 numbers and BAL-infiltrating cells were counted, and histological analyses were conducted (Figure 8G). As with the IL-33 model, higher lung resistance and a decrease in dynamic compliance were observed in mice receiving *A*. *alternata* rather than PBS, validating the *A*. *alternata*–induced AHR model. We found that treatment with a VISTA agonist compared with isotype control led to a significant reduction in lung resistance and improved dynamic compliance (Figure 8, H and I). This effect was corroborated by decreased numbers of pulmonary ILC2s (Figure 8J) and lower numbers of CD45^+^ cells and eosinophils in BAL fluid (Figure 8K). Additionally, lower levels of BAL IL-5, IL-6, and IL-13 (Figure 8L) as well as reduced airway epithelium thickening (Supplemental Figure 7, E and F) indicated that the lung inflammation was reduced in the group receiving the VISTA agonist. Collectively, these findings reveal that VISTA is a viable therapeutic target with potential to alleviate ILC2-mediated AHR in both IL-33– and allergen-induced models.

### Treatment with a VISTA agonist regulates human ILC2 function and AHR in humanized mice.

**Figure 9:**
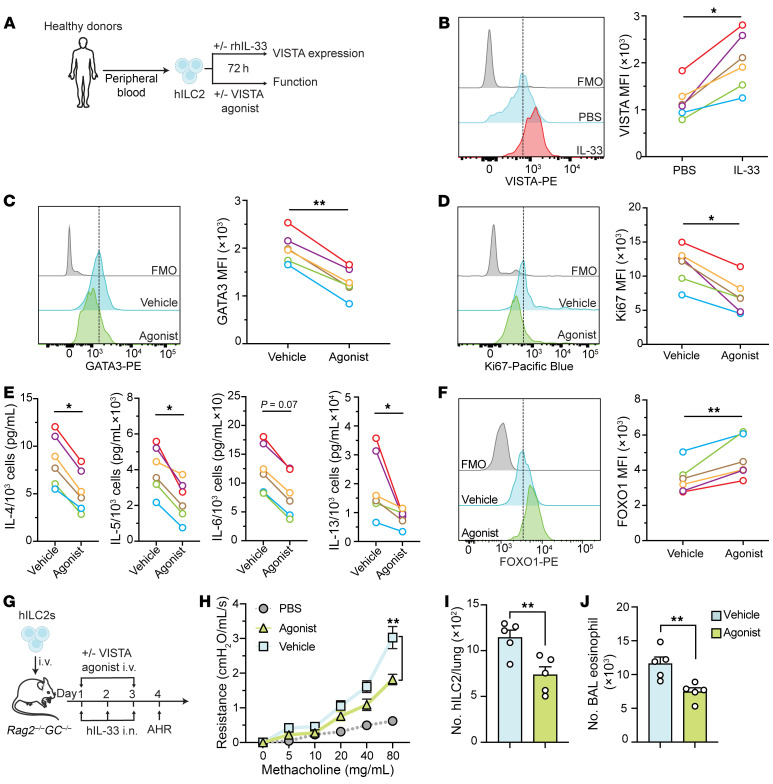
VISTA regulates human ILC2 function and alleviates ILC2-driven AHR in humanized mice. (**A**–**F**) Human peripheral ILC2s (hILC2s) were stimulated with or without rhIL-33 (100 ng/mL). (**B**) Representative plots of VISTA expression levels and corresponding quantification (as MFI). (**C**–**F**) hILC2s were treated with 5 μg/mL rhVSIG3 and vehicle control. (**C** and **D**) Representative plots of GATA-3 (**C**) and Ki67 (**D**) expression levels and corresponding quantification (as MFI). (**E**) Cytokine levels in hILC2 supernatant (per 10^3^ ILC2s). (**F**) Representative plots of FOXO1 expression levels and corresponding quantification (as MFI) following 1 hour treatment with rhVSIG3. (**G**–**J**) A total of 2 × 10^5^ hILC2s were transferred intravenously into *Rag2^–/–^GC^–/–^* mice, followed by intranasal challenge with rhIL-33. On days 1 and 3, mice intravenously received 2 mg/kg rhVSIG3 or vehicle. (**H**) Lung resistance in response to elevating doses of methacholine. (**I**) Total number of ILC2s per lung. (**J**) Total number of eosinophils in BAL fluid. Data are presented as mean + SEM and are representative of at least 2 independent experiments. Statistical significance was assessed using either 2-tailed Student’s *t* test (**B**–**F**, **I**, and **J**) or 1-way ANOVA followed by Tukey’s post hoc test (**H**); *P < 0.05, **P < 0.01.

Building on the observed regulatory effects of VISTA on ILC2s in mice, we sought to determine whether these findings could be translated to human ILC2 function and AHR. Fresh human ILC2s (hILC2s) were FACS-sorted from peripheral blood mononuclear cells of 6 healthy donors (Figure 9A). The hILC2s were identified as live cells expressing CD45, CD127, and CRTH2 and lacking lineage markers (Supplemental Figure 7G). Initially, we examined VISTA expression in hILC2s. Pure populations of hILC2s were cultured ex vivo with or without recombinant human IL-33 (rhIL-33) (Figure 9A). Flow cytometry analysis revealed that activation of hILC2s with IL-33 ex vivo resulted in the upregulation of VISTA protein on these cells (Figure 9B). Confirming our earlier observations in mice, VISTA expression was inducible in activated hILC2s isolated from all 6 donors. It has been well established that VSIG3 is a VISTA ligand that can bind to VISTA at physiological pH (14). To further investigate the physiological relevance of the VSIG3/VISTA pathway in the lung, we assessed the expression levels of VSIG3 (encoded by *Igsf11*) in the lungs of healthy and asthmatic patients (32). Notably, we found VSIG3 expressed in several cell types within the lung epithelium of both healthy individuals and asthma patients (Supplemental Figure 7H). Therefore, to further examine the impact of VISTA engagement on hILC2 activation, proliferation, and effector function, hILC2s from each donor were cultured with or without rhVSIG3 Fc protein as a VISTA agonist (Figure 9A) (9). Corroborating murine results, ex vivo treatment of hILC2s with VISTA agonist downregulated GATA-3 (Figure 9C) and Ki67 (Figure 9D), suggesting decreased hILC2 activation and proliferation. Moreover, VISTA ligation on hILC2s reduced the secretion levels of the ILC2 effector cytokines IL-4, IL-5, IL-6, and IL-13 in the culture supernatants across all 6 donors (Figure 9E). Next, we questioned whether the FOXO1-mediated effects of VISTA found in murine experiments could be corroborated in human ILC2s. Pure populations of hILC2s were treated with rhVSIG3, and the protein levels of FOXO1 were measured. Strikingly, VISTA ligation in hILC2s led to an elevated level of FOXO1 expression (Figure 9F). These findings consistently suggest that VISTA signaling upregulates the FOXO1 pathway in both mouse and human ILC2s.

To assess the in vivo effect of VISTA stimulation on hILC2 function, we evaluated whether VISTA stimulation could modulate the development of hILC2-dependent AHR and lung inflammation in a humanized mouse model (1). Pure populations of hILC2s were intravenously transferred into *Rag2^–/–^GC^–/–^* alymphoid mice. Host animals were then intranasally challenged with rhIL-33. The VISTA agonist or a corresponding vehicle was administered intravenously before the first and last intranasal challenges (Figure 9G). Initially, substantial lung resistance and a decrease in dynamic compliance were observed in mice receiving IL-33 rather than PBS, validating the humanized mice model (Figure 9H). Consistent with our findings in murine models, treatment with a VISTA agonist remarkably reduced lung resistance in comparison with vehicle control (Figure 9H). Although both groups initially received identical numbers of ILC2s, we observed a significant reduction in the number of hILC2s recovered from the lungs of mice treated with VISTA agonist compared with controls (Figure 9I). Additionally, BAL analysis from mice treated with the VISTA agonist showed fewer eosinophils, suggesting reduced hILC2-driven lung inflammation following VISTA stimulation (Figure 9J). Taken together, our results demonstrate that VISTA is expressed on human ILC2s and that stimulation with a VISTA agonist can reduce human ILC2–driven airway inflammation and AHR.

## Discussion

This study explores the role of VISTA, an inhibitory immune checkpoint, in regulating the function of pulmonary ILC2s and its therapeutic potential in treating ILC2-driven AHR. Our results reveal that VISTA expression is upregulated in activated pulmonary ILC2s and serves as a critical regulator of their activity through the FOXO1 pathway. VISTA-deficient ILC2s exhibit increased proliferation and effector function, leading to exacerbated AHR and lung inflammation. Conversely, VISTA agonism in both murine and humanized mice reduces ILC2 function and lung inflammation, presenting a promising therapeutic strategy for allergic AHR.

We first show that VISTA expression in pulmonary ILC2s is inducible at both transcriptomic and protein levels upon ILC2 activation. This inducibility is notable as it suggests that VISTA could be a therapeutic target to control lung inflammation. Recent studies have shown VISTA expression on various immune cells such as monocytes, macrophages, neutrophils, DCs, and T lymphocytes (11, 15, 16). Our study investigates the impact of VISTA on the functionality of ILC2s and its role in ILC2-mediated AHR using 2 distinct approaches: genetic deletion and pharmacological stimulation of VISTA. Our transcriptomic and protein analyses revealed that VISTA-deficient ILC2s displayed increased expression of ILC2 activation markers and type 2 cytokines, such as IL-5 and IL-13, which are crucial drivers of allergic inflammation. These results were validated in vivo using a VISTA-deficient mouse model, demonstrating that lack of VISTA led to excessive lung inflammation and worsened ILC2-mediated AHR. Since the IL-33 model is characterized as an acute ILC2-driven AHR, Th2 cells may not play a critical role in this context. However, given that VISTA-deficient mice lack VISTA on all cell types, we performed adoptive transfers of *Vsir^–/–^* ILC2s into mice lacking lymphoid cells. This model can specifically assess the impact of *Vsir^–/–^* ILC2s on ILC2-mediated AHR as the other cells express VISTA. Our findings revealed that VISTA deficiency in ILC2s alone is sufficient to exacerbate AHR development and lung inflammation. It was noteworthy that *Vsir^–/–^* ILC2s exhibited lower rates of apoptosis and increased proliferation rates, suggesting that VISTA-dependent higher survival and proliferation rates contributed to the expansion of the ILC2 population. Overall, our findings are consistent with previous studies that emphasize the critical role of VISTA in maintaining immune cell quiescence, showing that lack of VISTA increases susceptibility to various inflammatory disorders (9, 17, 18).

Previous studies from our laboratory and others have demonstrated that hyperactivated ILC2s exhibit high energy demands, which are primarily maintained through OXPHOS and FAO (33, 34). In the present study, our results suggest that VISTA regulates FAO and OXPHOS in ILC2s, as VISTA-deficient ILC2s exhibited increased FAO, TCA cycle, and oxidative respiration. The results of our transcriptomic analysis also revealed that genes encoding enzymes actively involved in the FAO are upregulated in VISTA-deficient ILC2s. This upregulation increases the predicted levels of acetyl-CoA, which in turn fuels the TCA cycle (35). Accordingly, we found enhanced TCA cycle signature in VISTA-deficient ILC2s at the transcriptomic level. The elevated oxidative respiration in ILC2s lacking VISTA was further validated using MitoTracker and TMRM staining and bioenergetic assays. Moreover, these cells exhibited increased fatty acid consumption and reduced fatty acid retention, as indicated by BODIPY assays. These findings highlight that VISTA-deficient ILC2s are metabolically more active, suggesting a potential role for VISTA in the modulation of ILC2 metabolism. The effect of VISTA on ILC2 metabolism was further confirmed using VISTA ligation on WT ILC2s. Our previous research has demonstrated that PD-1 restricts the effector functions of ILC2 by influencing their metabolic profile (5), suggesting that immune checkpoints might serve as metabolic regulators in ILC2s. Although the influence of VISTA on the metabolic activity of lymphoid cells has not been explored, a recent study in the myeloid lineage documented VISTA-mediated regulation of mitochondrial respiration in myeloid-derived suppressor cells (36). Further research is needed to clarify the impact of VISTA on metabolic alterations across other myeloid and lymphoid cell types.

Understanding the mechanism of action is crucial for unraveling the immunomodulatory functions of immune checkpoints. Thus, we conducted a comparison of the transcriptomic profiles between *Vsir^–/–^* and WT ILC2s, revealing enrichment of the FOXO1 signaling pathway as one of the prominent findings. Within this pathway, FOXO1-activating factors were increased while FOXO1-inhibiting factors were decreased. Using transcriptomic tools, we identified negative correlations between FOXO1 and several markers linked to ILC2 activation and proliferation within a weighted gene coexpression network analysis (WGCNA) module. WGCNA is often used as a powerful method in cell biology for understanding gene coexpression patterns and their relation to biological functions (37). We therefore hypothesized that VISTA regulates ILC2s via the FOXO1 pathway. Our subsequent protein analysis confirmed that VISTA deficiency correlates with reduced FOXO1 levels, and VISTA stimulation increased FOXO1 protein expression in ILC2s. We next investigated whether FOXO1 influences ILC2 proliferation and effector function using an established protocol with a FOXO1 inhibitor (26). Our results indicated that FOXO1 inhibition enhances ILC2 activation, leading to increased secretion of type 2 cytokines. Further validation of the regulatory role of FOXO1 in ILC2s was achieved by the use of FOXO1 activator as described by several groups before (26, 27). In line with our previous observations, FOXO1 activation resulted in reduced ILC2 activation and proliferation, thereby decreasing the secretion of type 2 cytokines. Taken together, our findings provide compelling evidence that VISTA modulates ILC2 effector function through the FOXO1 pathway.

FOXO1 is a member of the FOXO transcription factor family that has been shown to be influenced by factors from the tissue microenvironment (38). Multiple studies have highlighted the role of FOXO1 in immune regulation, demonstrating its ability to maintain the quiescence of naive T cells and restrain activation across various T cell subsets (39–41). Consistent with our findings, FOXO1 has been implicated in maintaining intestinal homeostasis in intestine-resident lymphocytes, as its downregulation has been linked to disrupted intestinal balance and exacerbated inflammation (42). Interestingly, ICOS signaling has been reported to deactivate FOXO1 (40). We previously showed that ICOS signaling enhances ILC2 effector function and exacerbates AHR (43). Given the role of ICOS as a costimulatory molecule, it is plausible that it may oppose VISTA by deactivating FOXO1, thereby potentially exacerbating AHR. However, the precise interplay between ICOS and VISTA in modulating FOXO1 and its impact on immune responses warrants further investigation. To further characterize the downstream effects of the AKT/FOXO1 pathway in ILC2s, we found that p65 and p52 are both increased in VISTA-deficient ILC2s. Previous studies have shown that FOXO1 acts as a negative upstream regulator of NF-κB (44, 45), suggesting that the role of VISTA via FOXO1 signaling may be linked to both canonical and non-canonical NF-κB signaling. Given that NF-κB complexes directly affect the TCA cycle (34, 46), our findings suggest that VISTA signaling, particularly via NF-κB, may cause the observed metabolic changes in ILC2s.

We next explored VISTA as a potential therapeutic target for attenuating ILC2-driven AHR by using an established anti–VISTA agonist antibody (17, 20, 25) and observed that engagement of VISTA significantly suppressed ILC2 function. To ensure specificity, we assessed the effects of the VISTA agonist on both WT and VISTA-deficient ILC2s. Importantly, while the VISTA agonist had a notable impact on WT ILC2s, it did not alter the function of ILC2s isolated from VISTA-deficient mice. This confirms that the agonist antibody regulates ILC2 functions in a VISTA-dependent manner. To address the effect of VISTA on lung inflammation and function, we administered VISTA agonist in mice and observed that it is capable of ameliorating IL-33–induced AHR and lung inflammation, as characterized by improved lung function, reduced pulmonary ILC2 numbers, and decreased inflammatory cytokine levels in BAL fluid. We further validated these results by assessing the VISTA engagement independent of adaptive immunity, using *Rag2*-deficient mice. Our findings demonstrated that the VISTA agonist effectively ameliorated ILC2-driven AHR and lung inflammation independent of adaptive immunity. Expanding our investigation to allergen-induced AHR, we used *A*. *alternata* as a clinically relevant allergen known for its serine protease activity, which induces alarmins such as IL-33 in the lungs (47). Consistent with our earlier models, we confirmed that the VISTA agonist effectively alleviates allergen-induced AHR driven by ILC2 activation. These results underscore the therapeutic potential of VISTA agonism for treating allergic asthma. Previous studies have reported the efficacy of VISTA agonists in preventing or improving mouse models of various inflammatory diseases (17, 18, 20, 25). Chen and colleagues recently reported reductions in cutaneous disease, autoantibodies, inflammatory cytokines, chemokines, and expansion of immune cells using anti–VISTA agonist antibody in a mouse model of lupus (17). In another study, treatment of acute-on-chronic liver failure (ACLF) mice with VISTA agonist suppressed T cell activation and cytokine production, considerably reducing mortality and liver inflammation in the mice (25). Additionally, it has been reported that anti–VISTA agonist antibody suppresses CD4^+^ T cell–mediated acute inflammation in a mouse model of acute hepatitis (18). VSIG3 and CD162 are identified as prominent VISTA ligands (12, 13). Among these, only VSIG3 can bind to VISTA at the physiological pH of the lungs (13, 14). Interestingly, VSIG3 is expressed in several cell types within the lung epithelium of both healthy individuals and asthma patients (32), suggesting it may be a more relevant ligand in this context.

Finally, to validate our murine findings in a human context, we investigated the expression and function of VISTA in human ILC2s. Similar to murine results, human ILC2s upregulated VISTA expression following activation. Consistent with our observations in murine studies, engagement of VISTA on human ILC2s reduced their activation, proliferation, and cytokine production. Interestingly, we found that VISTA ligation in human ILC2s also leads to FOXO1 upregulation, confirming the murine results in VISTA signaling. Furthermore, we used an ILC2 humanized murine model, established by our laboratory (34, 43, 48). In a humanized mouse model of ILC2-mediated AHR, VISTA agonist significantly reduced lung resistance and inflammation, highlighting its therapeutic potential in human ILC2–mediated allergic asthma. Interestingly, in clinical settings, VISTA upregulation has been reported in patients with systemic or discoid lupus erythematosus (17). Future investigations are needed to explore the expression pattern and functional implications of VISTA in asthma patients. Undoubtedly, understanding any potential correlation between VISTA expression and asthma severity could provide valuable insights for developing VISTA-targeted therapies to treat allergic asthma in humans.

In summary, our study highlights the critical role of VISTA in regulating ILC2 function and its therapeutic promise in allergic ILC2-mediated AHR. We demonstrated that VISTA engages the FOXO1 pathway to restrain ILC2 proliferation and effector function, thereby controlling ILC2-driven lung inflammation. These findings introduce new avenues for targeted immunotherapy in allergic asthma and other diseases involving ILC2s, leveraging VISTA agonist treatment to dampen pathological immune responses.

## Methods

### Sex as a biological variable.

Our study examined male and female animals, with similar findings reported for both sexes.

### Mouse models.

Wild-type (WT) BALB/cByJ (RRID:IMSR_JAX:001026), recombination-activating gene 2–deficient [*Rag2^–/–^*, C.B6(Cg)-*Rag2*tm1.1Cgn/J, RRID:IMSR_JAX:008448], and *Rag2*-deficient γ chain–deficient (*Rag2^–/–^GC^–/–^*, C;129S4-*Rag*2tm1.1FlvIl2rgtm1.1Flv/J, RRID:IMSR_JAX:014593) mice were obtained from The Jackson Laboratory. *Vsir^–/–^* [VISTA-KO, B6N.129S5(B6)-Vsirtm1Lex/Mmucd] mice were provided by Randolph J. Noelle and Louise Lines from Geisel School of Medicine at Dartmouth (Dartmouth College, Hanover, New Hampshire, USA). All mice used in experiments were on a BALB/c background and were sex-matched and aged between 5 and 8 weeks. They were housed and bred in a pathogen-free animal facility at the Keck School of Medicine, University of Southern California (USC), in accordance with protocols approved by the Institutional Animal Care and Use Committee.

### Pulmonary ILC2 isolation and ex vivo experiments.

Mice were intranasally challenged with 0.5 μg of rmIL-33 (BioLegend), rmIL-25 (BioLegend), or rmTSLP (R&D Systems) in 50 μL of PBS under anesthesia over 3 consecutive days. Control mice received only PBS. On the fourth day, the mice were euthanized, and transcardial perfusion was performed with PBS. Lungs were dissected and digested to single-cell suspension as previously described (49, 50). Pulmonary ILC2s were FACS-sorted to achieve a purity of greater than 95% on a FACSARIA III system. The ILC2s were identified based on the following markers: live cells, CD45^+^, lineage-negative (CD3ε, CD4, CD5, TCRβ, TCRγδ, CD45R/B220, CD335, CD11c, CD11b, Gr-1, FcεRIα, and Ter119), ST2^+^, and CD127^+^. Isolated ILC2s were cultured ex vivo following previous protocols (34). When specified, 10 μg/mL VISTA agonist antibody (MH5A, BioLegend), 10 μg/mL VISTA antagonist antibody (13F3, Bio X Cell), 100 nM FOXO1 inhibitor (AS1842856, MedChemExpress), or 1 μM FOXO1 activator (LOM612, MedChemExpress) with corresponding isotype control or vehicle (DMSO) was further added to cultures for the indicated times.

### Assessment of AHR and lung inflammation.

Mice were subjected to intranasal administration of 0.5 μg recombinant mouse IL-33 (rmIL-33), 100 μg of *A*. *alternata* (Greer Laboratories), or PBS, as detailed in prior studies (6). In certain experiments, mice were intraperitoneally injected with 5 mg/kg of anti–mouse VISTA agonist antibody (MH5A, BioLegend) or a corresponding isotype control in 200 μL PBS before each intranasal challenge. For adoptive transfer, 10^5^ ILC2s were intravenously injected into *Rag2^–/–^GC^–/–^* mice, and AHR was induced as previously described (34). AHR assessment, pulmonary ILC2 and BAL fluid cell analysis, cytokine measurement in BAL supernatant, and lung histological examination were performed according to the established protocols (7, 34).

### Flow cytometry.

Murine antibodies used in this study included FITC-conjugated antibodies targeting mouse lineage markers such as CD3ε (145-2C11), CD4 (GK1.5), CD5 (53-7.3), CD11b (M1/70), CD11c (N418), B220/CD45R (RA3-6B2), CD335/NKp46 (29A1.4), TCRβ (H57-597), TCRγδ (UC7-13D5), Gr-1 (RB6-8C5), Ter119 (TER-119), and FcεRIα (MAR-1), all sourced from BioLegend. Additional BioLegend murine antibodies included antibodies against CD127 (A7R34, PECy7), CD45 (30-F11, APCCy7/PECy7/FITC), CD11c (N418, APCCy7), SiglecF (S17007L, APC/APCCy7), Ly6G (1A8, APC/BV785), Ly6C (HK1.4, APCCy7/BV711), CD3ε (17A2, PerCP-Cy5.5), CD4 (GK1.5, BV421), TCR (H57-597, APC), Gr-1 (RB6-8C5, PerCP-Cy5.5/APC), IL-5 (TRFK5, PE/APC), VISTA/PD-1H (MIH63, PE/APC), I-A/I-E (M5/114.15.2, BV510), CD206 (C068C2, APC), CD88 (20/70, PE), CD64 (X54-5/7.1, PE), CD25 (PC61, BV421), CD90 (53-2.1, APC), and FOXO1 (W20064D, PE). Antibodies against mouse ST2 (RMST2-2, PerCP–eFluor 710), CD11b (M1/70, eFluor 450), Ki67 (SolA15, APC), GATA-3 (TWAJ, PE/eFluor 450), IL-13 (eBio13A, PE/APC), TCR (eBioGL3, PE), phospho-AKT (SDRNR, APC), phospho-BAD (BADS112-B9, PE), and BCL2 (10C4, PECy7) were purchased from Thermo Fisher Scientific. Anti–mouse SiglecF (E50-2440, PE), -ICOS (7E.17G9, PE), and -CD11c (HL3, PECy7) were procured from BD Biosciences. Anti-p65 (IC5078P, PE) was obtained from R&D Systems, and anti-p52 (C-5, PE) was purchased from Santa Cruz Biotechnology. Intranuclear staining was carried out as detailed previously (34). For intracellular staining, cells were stimulated 3 hours ex vivo with 50 μg/mL PMA, 500 μg/mL ionomycin (both from Sigma-Aldrich), and 1 μg/mL GolgiPlug (BD Biosciences) and then permeabilized using the BD Cytofix/Cytoperm kit according to the manufacturer’s protocol. For apoptosis staining, PE Annexin V (Thermo Fisher Scientific) and DAPI (Sigma-Aldrich) were added to cells according to the manufacturer’s guidelines. For caspase-3/7 staining, the CellEvent Caspase-3/7 Green Flow Cytometry Assay Kit (Thermo Fisher Scientific) was used according to the manufacturer’s protocol. When indicated, MitoTracker Green FM Dye, TMRM, BODIPY FL C_16_, and BODIPY^493/503^ (all from Thermo Fisher Scientific) were used according to the manufacturer instructions. Surface and intranuclear/intracellular antibodies were diluted at 1:200 and 1:50, respectively.

Human antibodies included a FITC-conjugated lineage cocktail targeting CD3 (UCHT1), CD14 (HCD14), CD16 (3G8), CD19 (HIB19), CD20 (2H7), and CD56 (HCD56). Additional FITC antibodies for lineage markers included CD1a (HI149), CD5 (L17F12), CD123 (6H6), CD235a (HI264), and FcεRIα (AER-37), all from BioLegend. Anti–human CD45 (HI30, APCCy7), -CD127 (A019D5, PECy7), -CRTH2 (BM16, PE), and -FOXO1 (W20064D, PE) were also purchased from BioLegend. PE-conjugated anti–human VISTA/PD-1H (MIH65.rMAb) was sourced from BD Biosciences. Anti–human GATA-3 (TWAJ, PE) and -Ki67 (20Raj1, eFluor 450) were purchased from Thermo Fisher Scientific. Dead cells were excluded using LIVE/DEAD Fixable Violet or Aqua cell stain kits (Thermo Fisher Scientific), and absolute cell numbers were calculated using CountBright absolute counting beads (Thermo Fisher Scientific). Cells were analyzed on a FACSCanto II system, and data were processed using FlowJo software version 10 (BD Biosciences).

### Mitochondrial bioenergetic assays.

The oxygen consumption rate was measured in real time using a T cell metabolic profiling kit and Seahorse Mini HS XF analyzer (Agilent) following the manufacturer’s protocol and as described in previous studies (34). Basal respiration, spare respiratory capacity, and ATP production rate were analyzed online using Agilent Seahorse Analytics.

### RNA sequencing and data analysis.

WT or *Vsir^–/–^* ILC2s were lysed using RLT buffer (QIAGEN), and total RNA was isolated using the MicroRNeasy kit (QIAGEN), according to the manufacturer’s protocol. The NextSeq 500 system (Illumina) was used for RNA sequencing (RNA-Seq) as detailed in our previous reports (1, 34). QIAGEN’s Ingenuity Pathway Analysis platform was used for comparative functional enrichment analysis. For single-cell RNA-Seq (scRNA-Seq) analysis, raw files were downloaded and processed as described previously (21). The initial quality control, preprocessing, and dimensionality reduction of scRNA-Seq data were performed using R programming language (version 4.3.0), the Seurat package (version 4.9.9), and the Markov Affinity-based Graph Imputation of Cells (MAGIC) algorithm. Data visualizations were performed using the R circlize package (version 0.4.15) and ggplot2 package (version 3.4.3).

### Weighted gene coexpression network analysis.

Bulk RNA-Seq data from VISTA-deficient and WT mice were analyzed using the weighted gene coexpression network analysis (WGCNA) package in R to construct gene coexpression networks and identify modules of highly coexpressed genes. A soft-thresholding power of 8 was selected based on the criterion of approximate scale-free topology, ensuring that the network adhered to the scale-free topology, a common characteristic of biological networks. Modules of coexpressed genes were identified using hierarchical clustering combined with dynamic tree cutting, with the dynamic tree cut method employed to segment the dendrogram produced by hierarchical clustering into distinct modules. To refine module definitions, modules with highly similar expression profiles were merged using a mergeCutHeight of 0.25.

### Human experiments and humanized mice.

Human ILC2s were isolated from peripheral blood mononuclear cells to a purity of greater than 95% using the autoMACS system (Miltenyi Biotec), followed by sorting with a FACSAria III cell sorter (BD Biosciences), as described previously. Human ILC2s were gated as CD45^+^, Lineage^–^ (CD3, CD5, CD14, CD16, CD19, CD20, CD56, CD235a, CD1a, CD123), CD127^+^, and CRTH2^+^. Isolated ILC2s were cultured at a density of 2 × 10^4^ cells/mL for 72 hours, as detailed in previous studies (1, 34). To agonize VISTA, 5 μg/mL recombinant human VSIG3 Fc protein (rhVSIG3; R&D Systems) and the corresponding vehicle control were added to the cultures. Humanized mouse model of AHR was carried out following an established protocol (34). Before the first and last intranasal challenges, rhVSIG3 (2 mg/kg; R&D Systems) or the corresponding vehicle was administered intravenously in 200 μL PBS.

### Cytokine quantification.

Cytokine levels in culture supernatants and BAL samples were quantified using either LEGENDplex Mouse Th Panel or LEGENDplex Human Th2 Panel (BioLegend) following the manufacturer’s instructions.

### Statistics.

All experiments were performed at least twice, and data are expressed as mean + SEM. Statistical analyses were performed using GraphPad Prism software (version 9.5.1). Comparisons between 2 groups were made using a 2-tailed Student’s *t* test for unpaired or paired data. For comparisons involving multiple groups, Tukey’s multiple-comparison 1-way ANOVA tests were used.

### Study approval.

Animal study was conducted in accordance with protocols approved by the USC Institutional Animal Care and Use Committee. The human study received approval from the USC Institutional Review Board and was conducted in compliance with the Declaration of Helsinki. Written informed consent was obtained from all participants prior to their involvement in the study.

### Data availability.

The data supporting the findings of this study are included in the article, supplemental materials, and Supporting Data Values file. RNA-Seq data were deposited in the NCBI’s Gene Expression Omnibus database (GSE271999). This study also used existing publicly available scRNA-Seq data (GSE102299). Schematic illustrations were obtained under an open-access license from Servier Medical Art.

## Author contributions

OA and MHK conceptualized the study. MHK, OA, ZMV, XL, BPH, YS, SS, PSJ, and KS devised methodology. OA acquired funding. MHK, SS, ZMV, and YS acquired human samples. OA supervised the study. MHK, XL, and ZMV analyzed data. MHK and OA wrote the original draft. MHK, OA, BPH, YS, ZMV, XL, SS, PSJ, and KS reviewed and edited the manuscript.

## Supplementary Material

Supplemental data

Supporting data values

## Version 1 01/02/2025

In-Press Preview

## Version 2 02/17/2025

Electronic publication
