# Supplementary material for: FOXO1 pathway activation by VISTA immune checkpoint restrains pulmonary ILC2 functions
Source: J Clin Invest. 2025 Jan 2;135(4):e184932. doi: 10.1172/JCI184932 (PMC11827891; doi:10.1172/JCI184932)
Supplement: Supplemental data [file jci-135-184932-s096.pdf]

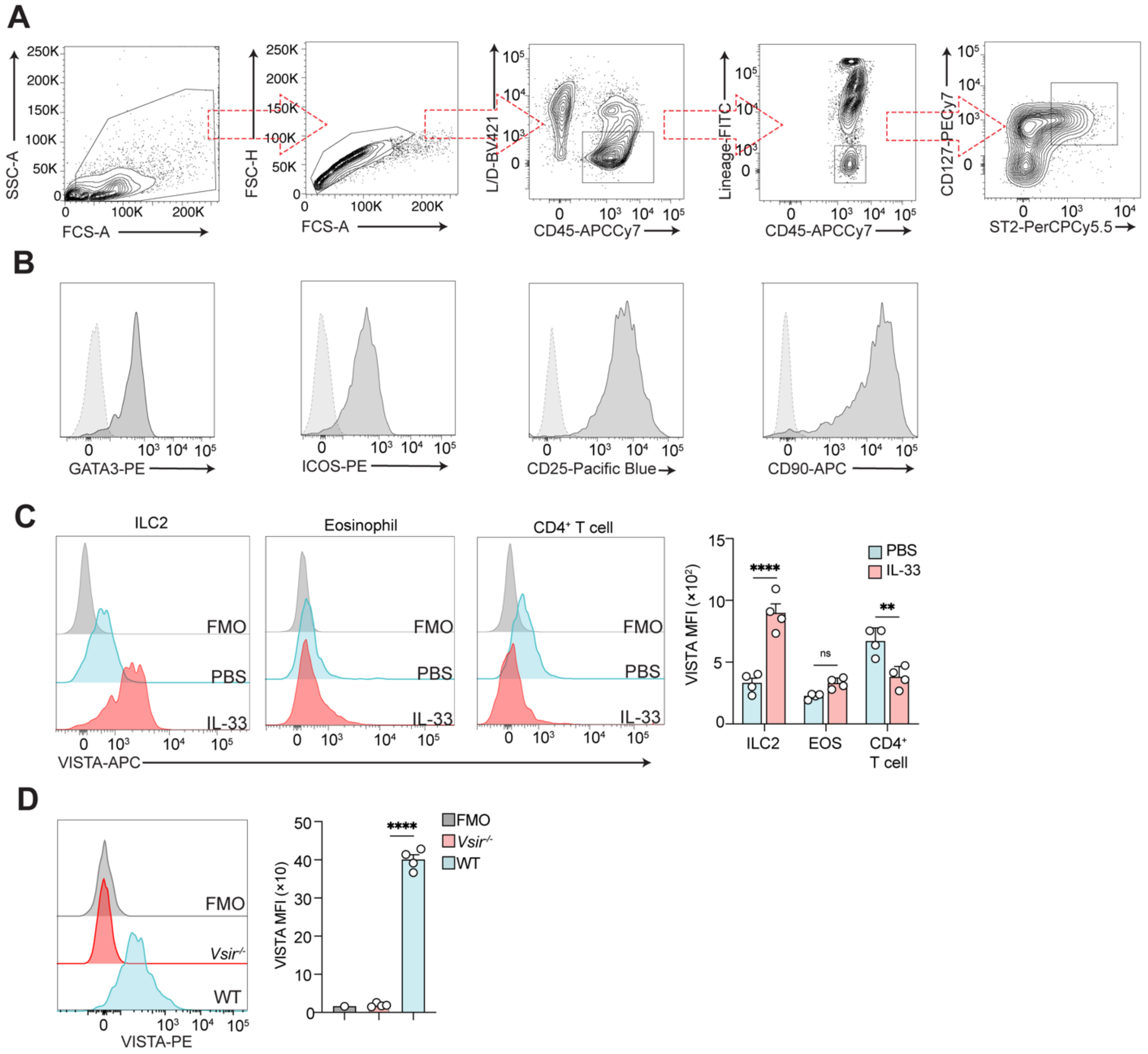

Supplemental Figure 1. Pulmonary ILC2 gating strategy and VISTA expression comparison. (A) Representative gating strategy for ILC2. ILC2s were gated as live single cells expressing CD45, CD127, and ST2, and lacking lineage markers (CD3ε, CD4, CD5, CD11b, CD11c, B220/CD45R, CD335/NKp46, TCRβ, TCRγδ, Gr-1, Ter119, and FcεRIa). **(B)** Isolated ILC2s were then assessed for the expression of GATA-3, ICOS, CD25 and CD90 as a quality control for cell sorting. Over 95% of isolated cells were positive for all markers. **(C)** WT mice were intranasally challenged with either 0.5 µg rmIL-33 or PBS for 3 days, On day 4, pulmonary ILC2s, eosinophils and $CD4^+$ T cells were stained for flow cytometry readout. Representative plots of VISTA expression levels and corresponding quantification (as MFI). **(D)** WT and VISTA-deficient mice were intranasally challenged with either 0.5 µg rmIL-33 or PBS for 3 days, On day 4, pulmonary ILC2s were stained for flow cytometry readout. Representative plots of VISTA expression levels and corresponding quantification (as MFI). Data are presented as mean+SEM and are representative of at least two independent experiments. Statistical significance was assessed using

either a two-tailed Student's t-test (C) or one-way ANOVA followed by Tukey's post-hoc test (D); $^{**}p < 0.01$, $^{****}p < 0.0001$.

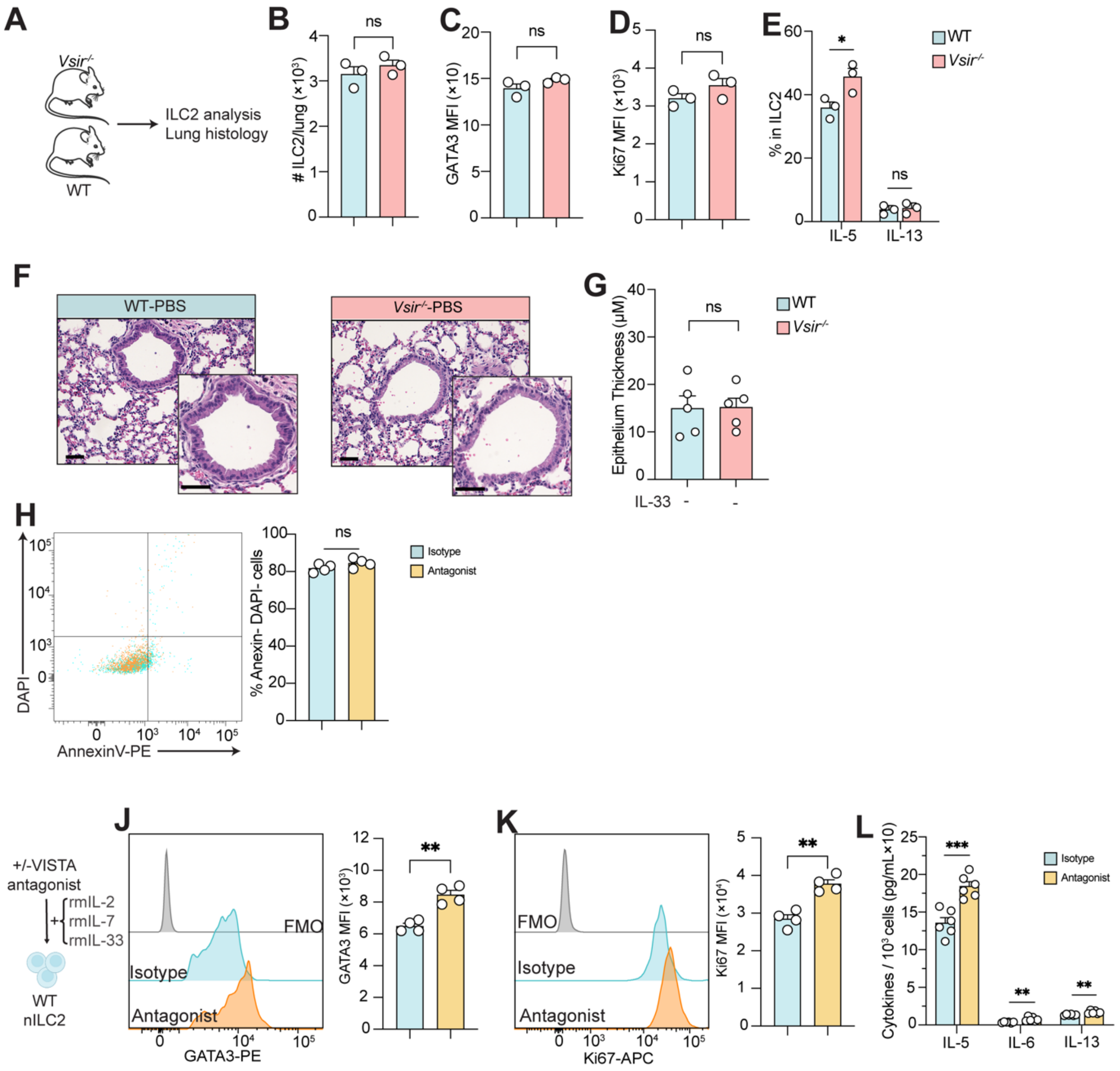

Supplemental Figure 2. Effects of VISTA blocking on ILC2s.

(A-G) Pulmonary ILC2s number (B), GATA-3 (C) and Ki67 (D) expression levels, IL-5+ ILC2s and IL13+ ILC2s (E), and lung epithelium thickness (F-G) were compared between WT and VISTA-deficient mice at steady state. **(H)** Representative dot plot for annexinV-DAPI apoptosis assay and corresponding quantitation of live cell frequency following treatment with anti-VISTA antagonist antibody (13F3). **(I-L)** WT naïve ILC2s were cultured with rmIL-2, rmIL-7 and rm-IL33 in the presence of VISTA antagonist antibody (13F3) or isotype control. **(J-K)** Representative plots of GATA-3 (J) and Ki67 (K) expression levels and corresponding quantification (as MFI). **(L)** Quantitation of IL-5, IL-6 and IL-13 levels in cell supernatant (per 10^3 ILC2s). Data are presented as mean+SEM and are representative of at least two independent experiments. Statistical significance was assessed using a two-tailed Student's t-test; *p < 0.05, **p < 0.01, ***p < 0.001.

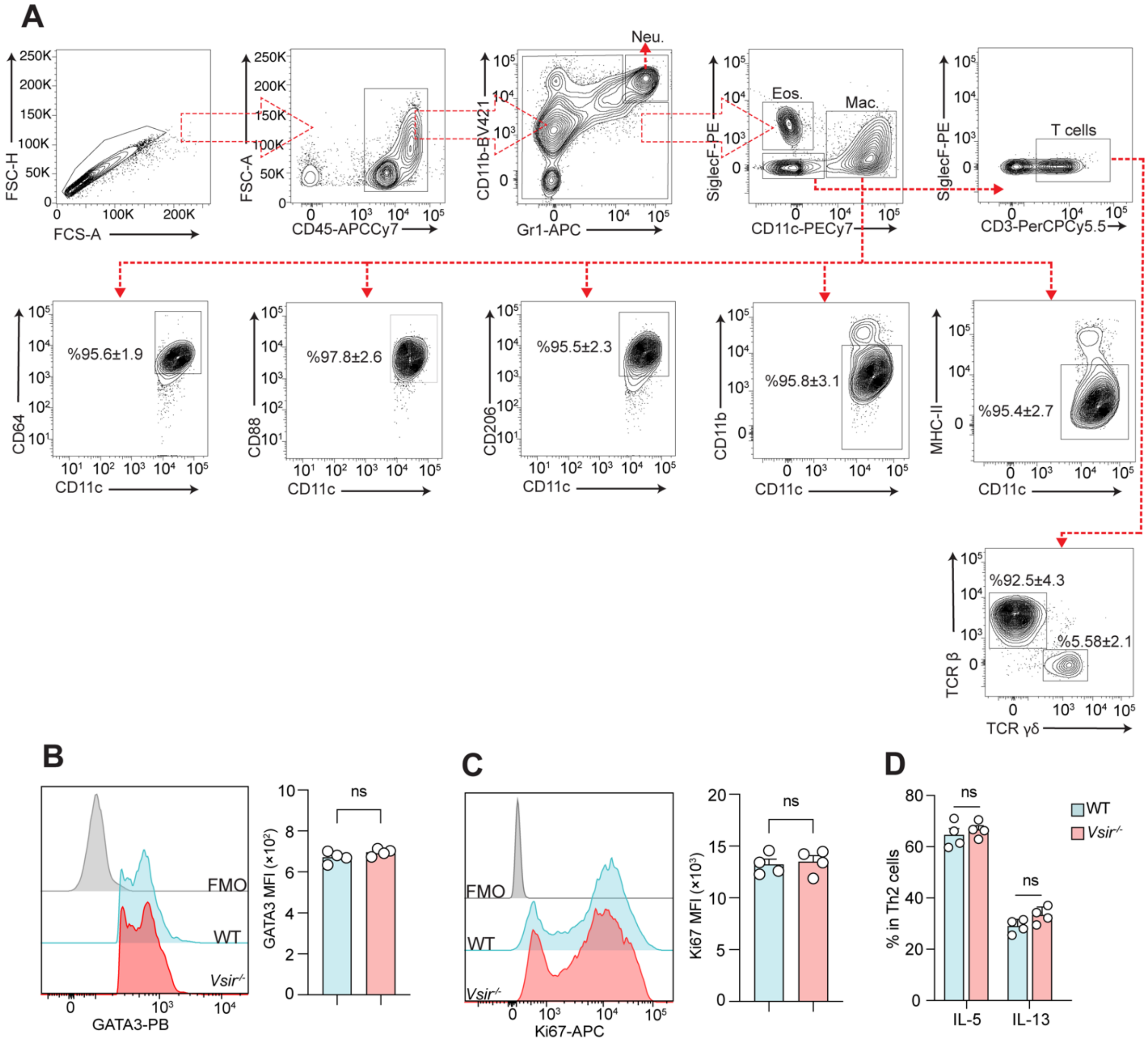

Supplemental Figure 3. BAL immune cell gating strategy and T$_H$2 activation comparison. **(A)** Representative gating strategy for leukocytes (CD45$^+$), eosinophils (CD45$^+$, Gr-1$^-$, CD11c$^-$, SiglecF$^+$), neutrophils (CD45$^+$, SiglecF$^-$, Gr1$^+$, CD11b$^+$), macrophages (CD45$^+$, Gr1$^-$, CD11c$^+$), and T cells (CD45$^+$, CD3$^+$). Macrophages were further assessed for CD64, CD88, CD206, CD11b, MHC-II, and over 95% were CD64$^+$, CD88$^+$, CD206$^+$, CD11b$^{low/-}$, MHC-II $^{low/-}$. T cells were further assessed for TCR β and TCR λδ. **(B-D)** WT and VISTA-deficient mice were intranasally challenged with 0.5 µg rmIL-33 or PBS during 3 days. On day 4, pulmonary T$_H$2 cells were assessed for activation status. Representative plots of GATA-3 (B) and Ki67 (C) expression levels and corresponding quantification (as MFI). **(D)** Frequency of IL-5$^+$ cells and IL-3$^+$ cells in T$_H$2 cells. Data are presented as mean+SEM and are representative of at least two independent experiments. Statistical significance was assessed using a two-tailed Student's t-test; ns. non-significant;

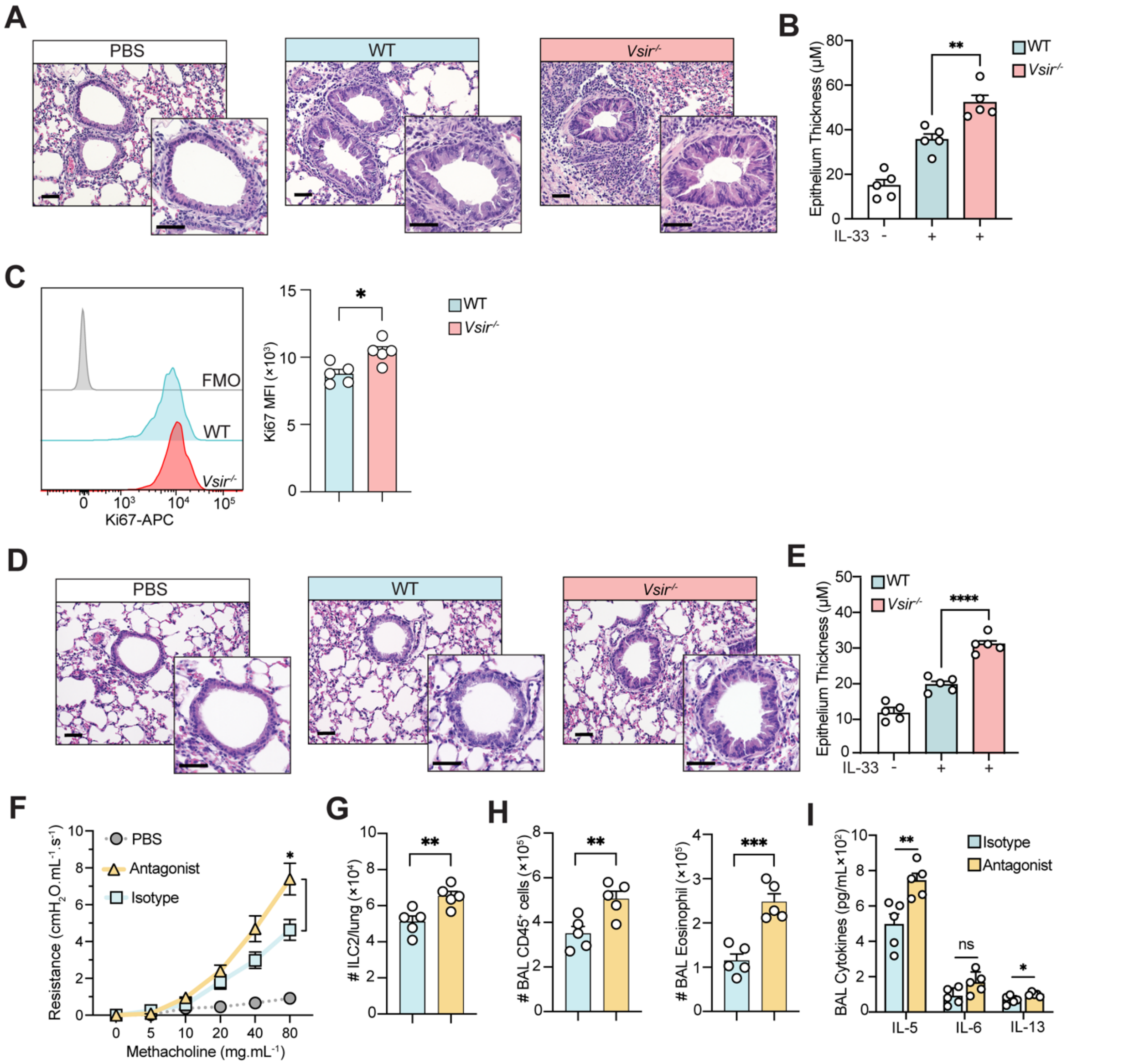

Supplemental Figure 4. Histological examination, Ki67 expression, and in vivo VISTA blocking.

(A-B) WT and VISTA-deficient mice were intranasally challenged with 0.5 µg rmIL-33 or PBS during 3 days. On day 4, lung histology was examined. **(A)** Representative hematoxylin and eosin (H&E) stained lung sections; scale bars=50µm. **(B)** Quantification of alveolar epithelium thickness. **(C-E)** WT and VISTA-deficient mice received 3 days of intranasal rm-IL33, and pulmonary aILC2s were then isolated. Cohorts of *Rag*$^{-/-}$*GC*$^{-/-}$ alymphoid mice were intravenously injected with 10^5 aILC2s isolated from WT or VISTA-deficient mice. Recipient mice were intranasally challenged with 1 µg rmIL-33 or PBS for 3 days. On day 4, Ki67 expression levels of pulmonary ILC2s and lung histology was examined. **(C)** Representative plots of Ki67 expression levels and corresponding quantification (as MFI). **(D)** Representative hematoxylin and eosin (H&E) stained lung sections; scale bars=50µm. **(E)** Quantification of alveolar epithelium thickness. **(F-I)** WT mice were intraperitoneally injected with 5 mg/kg of anti-VISTA antagonist antibody or isotype control, followed by intranasal challenge with rmIL-33 or PBS. **(F)** Lung

resistance in response to elevating doses of methacholine. **(G)** Total number of ILC2s per lung. **(H)** Total number of $CD45^+$ cells and eosinophils in BAL fluid. **(I)** Cytokine levels in the BAL fluid. Data are presented as mean+SEM and are representative of at least two independent experiments. Statistical significance was assessed using either a two-tailed Student's t-test (C and G-I) or a one-way ANOVA followed by Tukey's post-hoc test (B, E and F); *p < 0.05, **p < 0.01, ****p < 0.0001.

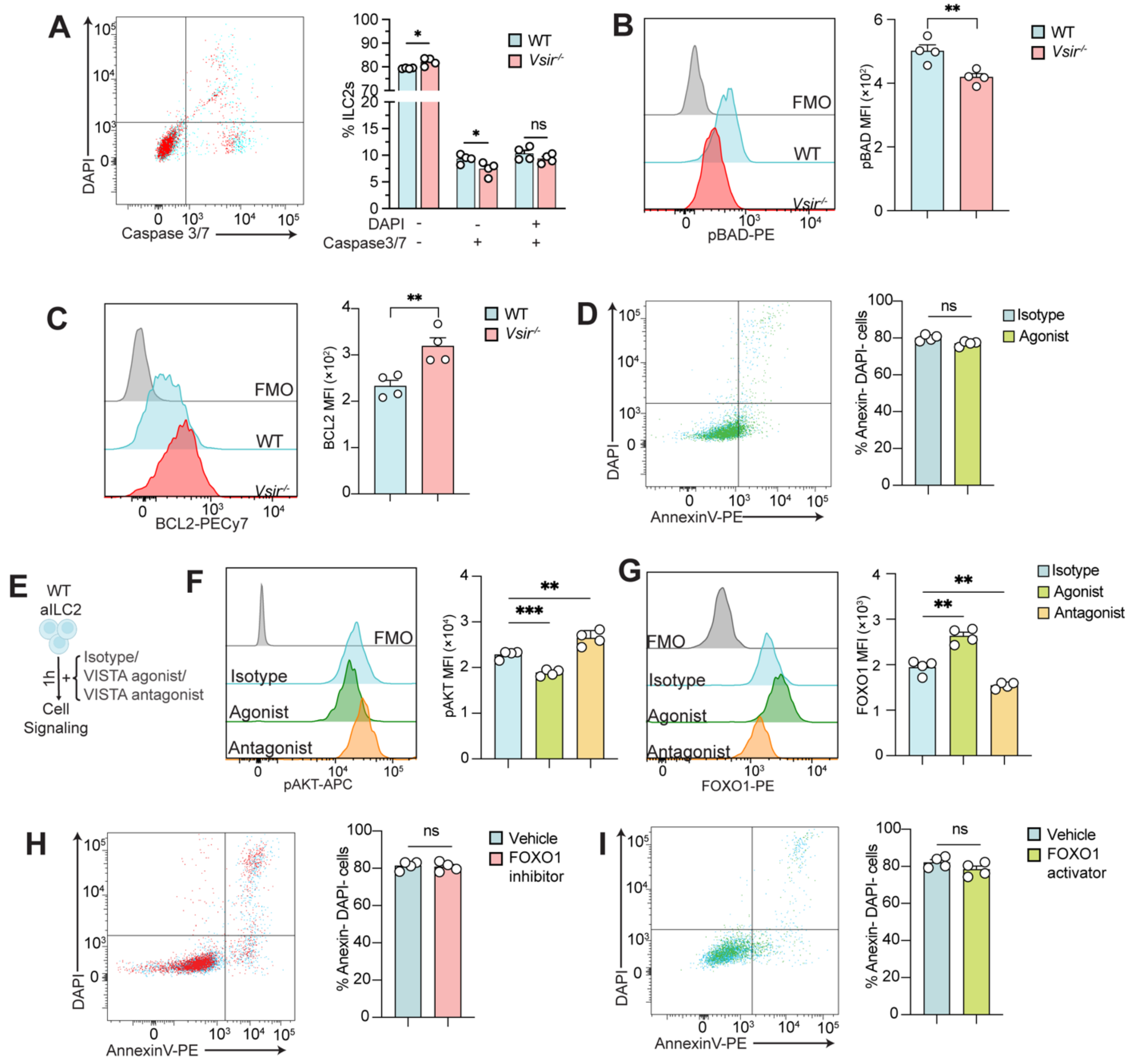

Supplemental Figure 5. Apoptosis assay and FOXO1 expression following VISTA agonist/antagonist treatment.

(A-C) WT and *Vsir*$^{-/-}$ ILC2s were stained with CellEvent Caspase-3/7 Green reagent and DAPI, anti-phosphorylated BAD (pBAD) antibody, or anti-BCL2 antibody. **(A)** Representative dot plot for Caspase3/7-DAPI staining and corresponding quantifications as frequency. **(B-C)** Representative plots of pBAD (B) and BCL2 (C) expression levels and corresponding quantification (as MFI). **(D)** Representative dot plot for annexinV-DAPI apoptosis assay and corresponding quantitation of live cell frequency following treatment with VISTA agonist. **(E-G)** WT naïve ILC2s were ex vivo stimulated with rmIL-33. Ex vivo activated ILC2s were then treated with either VISTA agonist, antagonist, or isotype for 1 hour. **(F-G)** Representative plots of pAKT (F) and FOXO1 (G) expression levels and corresponding quantification (as MFI). **(H-I)** Representative dot plot for annexinV-DAPI apoptosis assay and corresponding quantitation of live cell frequency following treatment with FOXO1 inhibitor (H), and FOXO1 activator (I). Data are presented as mean+SEM and are representative of at least two independent experiments. Statistical

significance was assessed using either a two-tailed Student's t-test (A-D and H-I) or a one-way ANOVA followed by Tukey's post-hoc test (F-G); *$p < 0.05$, **$p < 0.01$, ***$p < 0.001$.

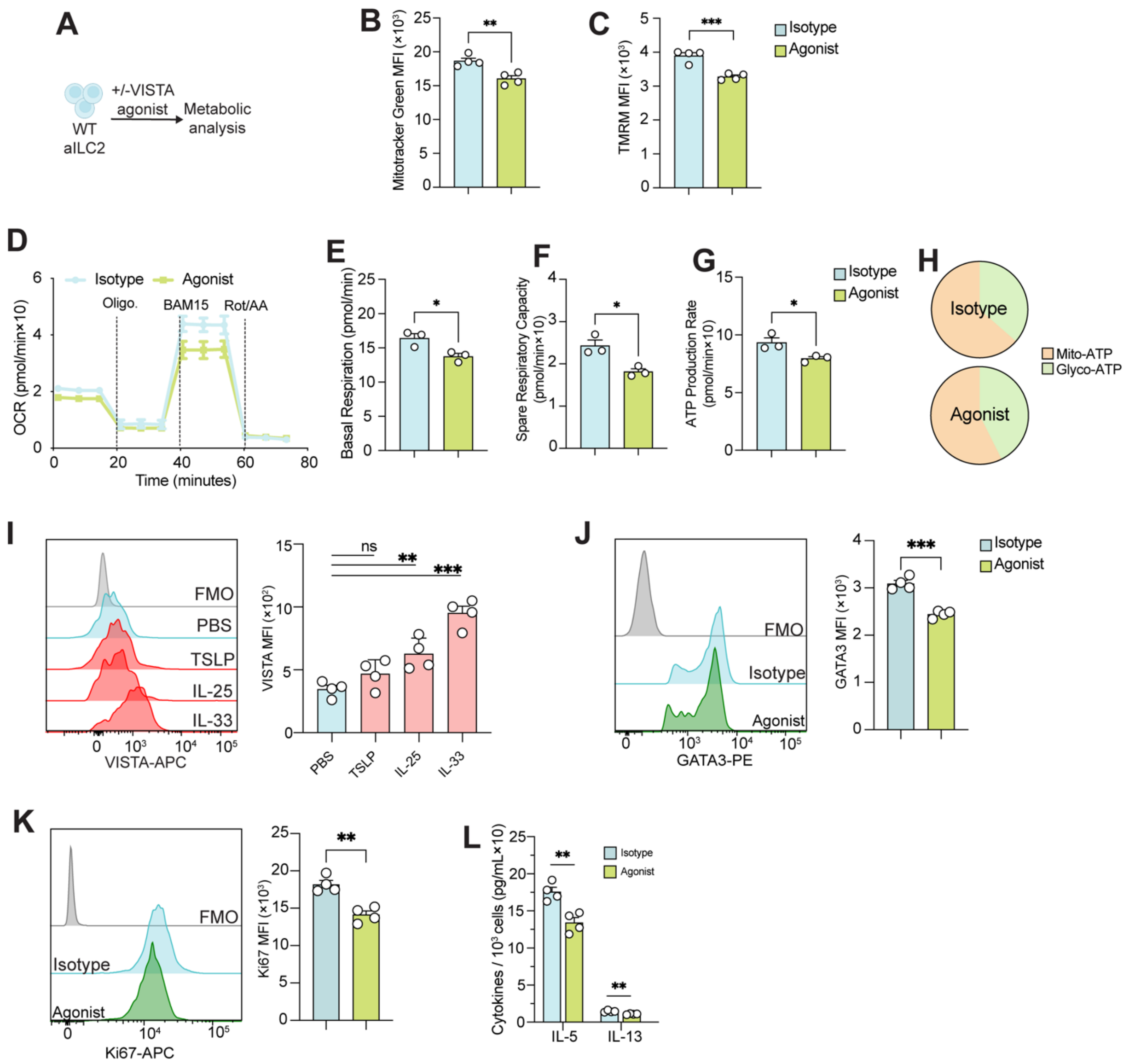

Supplemental Figure 6. Effect of VISTA ligation on metabolism and function of ILC2s. (A-H) WT aILC2s were treated ex vivo with VISTA agonist and metabolic assessment were performed. (B-C) Representative plots of MitoTracker Green (B) and TMRM (C) expression levels and corresponding quantification (as MFI). **(D-H)** Mitochondrial respiratory profile. **(D)** Oxygen consumption rate (OCR) in response to Oligomycin, BAM15, and Rotenone + antimycin A sequential injections. **(E-H)** Basal respiration (E), spare respiratory capacity (F), ATP production rate (G), mitochondrial (mito-ATP) and glycolytic (glycol-ATP) ATP production (H). **(I-L)** WT mice were intranasally challenged with 0.5 µg of either rmIL-33, rmIL-25, rmTSLP or PBS during 3 days. On day 4, pulmonary ILC2s were stained for either FACS-sorting or flow cytometry readout. **(I)** Representative plot of VISTA expression levels and corresponding quantification (as MFI). **(J-L)** Pulmonary IL-25-activated ILC2s were FACS-sorted and cultured with rmIL-2 and rmIL-7 in the presence of either VISTA agonist or isotype. **(J-K)** Representative plots of GATA-3 (J) and Ki67 (K) expression levels and corresponding quantification (as MFI). **(L)** Quantification of IL-5 and IL-13 levels in ILC2 supernatant (per 10^3 ILC2s) following addition of VISTA agonist.

Data are presented as mean+SEM and are representative of at least two independent experiments. Statistical significance was assessed using either a two-tailed Student's t-test (B-G and J-L) or a one-way ANOVA followed by Tukey’s post-hoc test (I);*$p < 0.05$, ** < 0.01, ***$p < 0.001$, ns: non-significant.

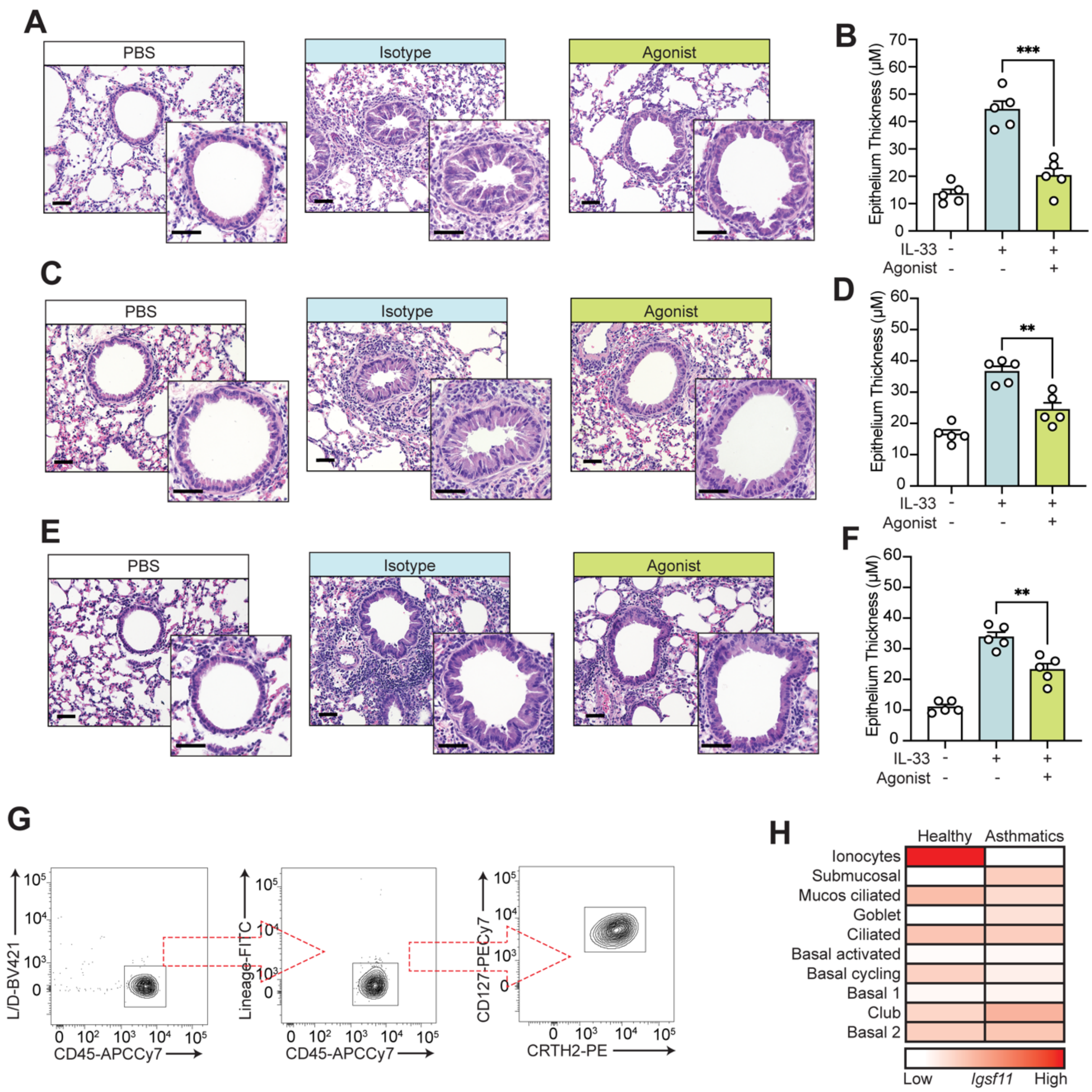

Supplemental Figure 7. Pulmonary histological examination and human ILC2 gating strategy. (A-B) WT mice were intraperitoneally injected with 5 mg/kg of anti-mouse VISTA agonist antibody or isotype control, followed by intranasal challenge with 0.5 µg rmIL-33 or PBS for 3 days. On day 4, lung histology was examined. **(A)** Representative hematoxylin and eosin (H&E) stained lung sections; scale bars=50µm. **(B)** Quantification of alveolar epithelium thickness. **(C-D)** *Rag2*$^{-/-}$ mice were intraperitoneally injected with 5 mg/kg of anti-mouse VISTA agonist antibody or isotype control, followed by intranasal challenge with 0.5 µg rmIL-33 or PBS for 3 days. On day 4, lung histology was examined. **(C)** Representative hematoxylin and eosin (H&E) stained lung sections; scale bars=50µm. **(D)** Quantification of alveolar epithelium thickness. **(E-F)** *Rag2*$^{-/-}$ mice were intraperitoneally injected with 5 mg/kg of anti-mouse VISTA agonist antibody or isotype control, followed by intranasal challenge with 100 µg of *A. alternata* or PBS for 4 days. On day 5, lung histology was examined. **(E)** Representative hematoxylin and eosin (H&E) stained lung sections; scale bars=50µm. **(F)** Quantification of alveolar epithelium thickness. **(G)** Representative gating strategy for human ILC2. Human ILC2s were gated as live

single cells expressing CD45, CD127, and CRTH2, and lacking lineage markers (CD3, CD5, CD14, CD16, CD19, CD20, CD56, CD235a, CD1a, CD123). **(H)** The expression of VSIG3 (*Igsf11*) within lungs of healthy subjects versus asthma patients. Data were analyzed from a previously published and publicly available data set (PMID:31209336). Data are presented as mean+SEM and are representative of at least two independent experiments. Statistical significance was assessed using one-way ANOVA followed by Tukey's post-hoc test; $^{**}p < 0.01$, $^{***}p < 0.001$;

Supplemental Table 1. Top upregulated genes in *Vsir*^{high} ILC2s.

1	*Vsir*	7	*Rin2*	13	*Clec1b*	19	*Adap2os*	25	*Rnase2a*	31	*Clec4b1*
2	*Col6a2*	8	*Plxna4os1*	14	*Thrb*	20	*Rnf150*	26	*Mcf2l*	32	*Mtus1*
3	*Tfec*	9	*Ms4a7*	15	*Sdk2*	21	*Msr1*	27	*Ctf2*	33	*Soga1*
4	*Adgrl4*	10	*Myof*	16	*Adap2*	22	*Fmnl2*	28	*Hbq1b*	34	*Kcnj8*
5	*Hp*	11	*Mylk*	17	*Zdbf2*	23	*Nsg2*	29	*Podxl*	35	*Rab30*
6	*Cd33*	12	*Sirpb1c*	18	*Tmem204*	24	*Wfdc17*	30	*Serpinb9b*	36	*Psd2*

Activated ILC2s were stratified into *Vsir*^{high} and *Vsir*^{low}, based on their *Vsir* transcript levels, using the 25% of the population with the lowest expression levels (Q1) as *Vsir*^{low} and the 25% with highest expression (Q4) as *Vsir*^{high} cells. Top genes most upregulated in *Vsir*^{high} population, based on statistically significant fold changes compared to *Vsir*^{low} population.
